# Ancient DNA Analysis of 8000 B.C. Near Eastern Farmers Supports an Early Neolithic Pioneer Maritime Colonization of Mainland Europe through Cyprus and the Aegean Islands

**DOI:** 10.1371/journal.pgen.1004401

**Published:** 2014-06-05

**Authors:** Eva Fernández, Alejandro Pérez-Pérez, Cristina Gamba, Eva Prats, Pedro Cuesta, Josep Anfruns, Miquel Molist, Eduardo Arroyo-Pardo, Daniel Turbón

**Affiliations:** 1 Research Centre in Evolutionary Anthropology and Paleoecology, Liverpool John Moores University, Liverpool, United Kingdom; 2 Laboratorio de Genética Forense y Genética de Poblaciones, Dpto. Toxicología y Legislación Sanitaria, Facultad de Medicina, Universidad Complutense de Madrid, Madrid, Spain; 3 Dpto. Biología Animal-Unidad de Antropología, Facultad de Biología, Universitat de Barcelona, Barcelona, Spain; 4 Centro de Investigación y Desarrollo, Consejo Superior de Investigaciones Científicas, Barcelona, Spain; 5 Dpto. de Apoyo a la Investigación, Servicios informáticos de la Universidad Complutense de Madrid, Madrid, Spain; 6 Dep. Prehistoria, Facultad de Filosofía y Letras, Universitat Autónoma de Barcelona, Bellaterra, Barcelona, Spain; Dartmouth College, United States of America

## Abstract

The genetic impact associated to the Neolithic spread in Europe has been widely debated over the last 20 years. Within this context, ancient DNA studies have provided a more reliable picture by directly analyzing the protagonist populations at different regions in Europe. However, the lack of available data from the original Near Eastern farmers has limited the achieved conclusions, preventing the formulation of continental models of Neolithic expansion. Here we address this issue by presenting mitochondrial DNA data of the original Near-Eastern Neolithic communities with the aim of providing the adequate background for the interpretation of Neolithic genetic data from European samples. Sixty-three skeletons from the Pre Pottery Neolithic B (PPNB) sites of Tell Halula, Tell Ramad and Dja'de El Mughara dating between 8,700–6,600 cal. B.C. were analyzed, and 15 validated mitochondrial DNA profiles were recovered. In order to estimate the demographic contribution of the first farmers to both Central European and Western Mediterranean Neolithic cultures, haplotype and haplogroup diversities in the PPNB sample were compared using phylogeographic and population genetic analyses to available ancient DNA data from human remains belonging to the *Linearbandkeramik-Alföldi Vonaldiszes Kerámia* and Cardial/Epicardial cultures. We also searched for possible signatures of the original Neolithic expansion over the modern Near Eastern and South European genetic pools, and tried to infer possible routes of expansion by comparing the obtained results to a database of 60 modern populations from both regions. Comparisons performed among the 3 ancient datasets allowed us to identify K and N-derived mitochondrial DNA haplogroups as potential markers of the Neolithic expansion, whose genetic signature would have reached both the Iberian coasts and the Central European plain. Moreover, the observed genetic affinities between the PPNB samples and the modern populations of Cyprus and Crete seem to suggest that the Neolithic was first introduced into Europe through pioneer seafaring colonization.

## Author Summary

Since the original human expansions out of Africa 200,000 years ago, different prehistoric and historic migration events have taken place in Europe. Considering that the movement of the people implies a consequent movement of their genes, it is possible to estimate the impact of these migrations through the genetic analysis of human populations. Agricultural and husbandry practices originated 10,000 years ago in a region of the Near East known as the Fertile Crescent. According to the archaeological record this phenomenon, known as “Neolithic”, rapidly expanded from these territories into Europe. However, whether this diffusion was accompanied or not by human migrations is greatly debated. In the present work, mitochondrial DNA –a type of maternally inherited DNA located in the cell cytoplasm- from the first Near Eastern Neolithic populations was recovered and compared to available data from other Neolithic populations in Europe and also to modern populations from South Eastern Europe and the Near East. The obtained results show that substantial human migrations were involved in the Neolithic spread and suggest that the first Neolithic farmers entered Europe following a maritime route through Cyprus and the Aegean Islands.

## Introduction

The term “Neolithic” refers to the profound cultural and technical changes that accompanied the transition from a hunter-gatherer subsistence economy to an agro-pastoral producing system [1]. The first Neolithic societies originated 12 to 10 thousand years ago in a region of the Near East traditionally known as the “Fertile Crescent” [2]. From this region the Neolithic technology rapidly expanded to Anatolia reaching the rest of Europe in less than 3,000 years by following two main routes linked to different archaeological cultural complexes. The Danubian route, associated to the *Linearbandkeramic* (LBK) cultural complex, brought the Neolithic to the central European plains and from there to the British Islands and Scandinavia (Funnel Beaker Cultural Complex) while the Mediterranean one, associated to the *Cardial-Impressa* cultural complex, spread it along the Mediterranean coast up to the Atlantic façade of Iberia [3].

The nature of the diffusion of the Neolithic and the possible demographic input associated to it have been widely debated. In this regard, two extreme hypotheses representing opposite views have been formulated: the demic diffusion model (DDM) and the cultural diffusion model (CDM) [1], [2], [4], [5]. The former stands up for a “wave of advance” of Neolithic immigrants with subsequent genetic replacement of the hunter-gatherer (Mesolithic) populations while the latter proposes a cultural adoption of Neolithic practices from local populations, implying a genetic continuity since the Palaeolithic. Moreover, integrationist models that involve a different extent of interaction between immigrants and local hunter-gatherers while considering the effect of geographic barriers and agricultural boundary zones, have been also used to explain the transition to the Neolithic process at a more local scale [6].

Genetic analyses from modern and ancient populations have contributed extensively to this debate providing discordant results. Principal component analysis and spatial autocorrelation of allele frequencies of “classic” genetic markers in modern European populations showed a South East to North West cline compatible with a Neolithic DDM. The Neolithic contribution to the modern genetic pool was estimated in this case to be around 27% [7]. The frequency distribution of Y chromosome polymorphisms displayed a similar pattern and haplogroups F*, E3b, G and J2, representing a 22% of extant lineages, were initially identified as the main contributors of the Neolithic spread [8], [9]. However, the analysis of the geographic distribution of the microsatellite diversity of the allegedly Paleolithic haplogroup R1b1b2, has been recently reinterpreted as a signal of substantial demic diffusion [10]. Phylogeographic analyses of another haploid marker, the mitochondrial DNA (mtDNA), in Europe and the Near East initially supported a limited Neolithic genetic contribution of around 9–12% in the Mediterranean and 15–22% in Central Europe [11]. Molecular dating and founder analyses identified then mtDNA haplogroups J, T1 and U3 as the main genetic markers of this expansion, with probable contributions of some other lineages from clusters H and W [12]. However, recent analysis of complete mtDNA sequences from the same region has pictured contradicting results depending on the analysis performed, from all mtDNA haplogroup expansions predating the Neolithic [13] to Neolithic expansions of mtDNA haplogroup H [14].

In the light of these results, the usefulness of modern genetic variability to reconstruct the Neolithic dynamics in Europe has been questioned [15], [16]. First of all, a certain level of genetic differentiation between hunter-gatherers and Near Eastern farmers has to be assumed in order to detect differences between both groups. Secondly, the existence of SE-NW clinal patterns in Europe may reflect the accumulation of small migrations entering the continent rather than a single migratory event [17]. Finally, original population substructure and subsequent processes of genetic drift and founder effects can introduce errors into the estimation of coalescence dates of mitochondrial and Y chromosome haplogroups [18]. In this regard, recent diachronic aDNA analyses of Central European populations have documented a fluctuation in haplogroup frequencies as a result of population bottlenecks and post-Neolithic migratory events [19], [20]. Besides, these estimated haplogroup dates do not necessarily correspond to the time of arrival of the lineages to the region [21]. As a result, the misidentification of genetic variants associated to the Neolithic spread and the effect of post-Neolithic expansions in the genetic make-up of Europe could have introduced important biases in the estimations of the Neolithic component of the European gene pool producing misleading conclusions [22].

During the last decade, ancient DNA analyses of Neolithic populations have provided a more reliable picture of the Neolithic transition process at a local scale. Studies have concentrated at the two edges of the two routes of the Neolithic wave of advance: Central/Northern Europe and the Iberian Peninsula/Southern France. In Central Europe and Scandinavia a DDM has been proposed to explain the observed genetic discontinuity between hunter-gatherers and the first farmer populations [19], [23]–[26]. However, recent analyses have suggested the coexistence of genetically distinct hunter-gatherer and farmer groups during several millennia at the same archaeological site, suggesting that the genetic replacement of hunter-gatherers populations was not complete [20]. In North Eastern Iberia and Southern France contradictory interpretations have been proposed to explain the nature of the Mesolithic-Neolithic transition process. On one hand, mtDNA studies of Cardial Neolithic remains seem to favor a pioneer Near Eastern colonization of North Eastern Spain [27], [28]. On the other hand, mtDNA results of Epicardial, Middle and Late Neolithic populations have been interpreted as a signal of pre-Neolithic legacy [29]–[31]. Dating and cultural differences between the studied groups, the effect of genetic drift at the beginning of the Neolithic and differences in the methods of analysis used (model-based statistical inference *vs* assignment of mtDNA haplogroup dating categories respectively) could be responsible of the observed differences [12], [27]. Moreover, the Y chromosome diversity of the Epicardial and Late Neolithic datasets has also shown a predominantly Near Eastern influence, suggesting that males and females might have played a differential role in the Neolithic dissemination process [16], [30], [31].

In this framework, the knowledge of the original Neolithic genetic pool in the Near East seems essential to correctly identify the variants associated to the Neolithic spread and also to provide a more global picture of the Neolithic dynamics in Europe.

**Figure 1:**
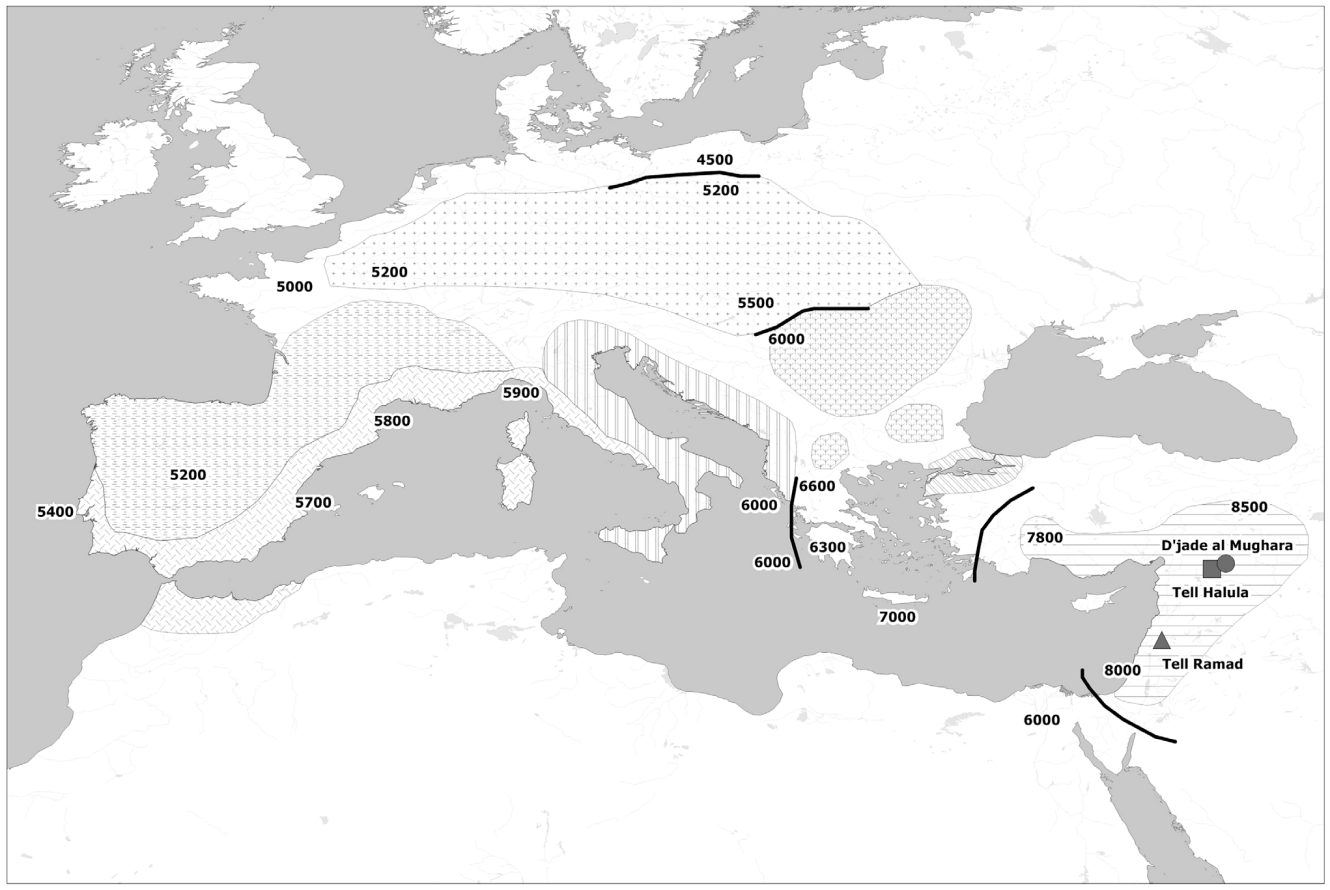
Map of the spread of Neolithic farming cultures in Europe. Shadings represent isochronous Neolithic archaeological cultures and black lines frontier zones between them. Analyzed sites in the Fertile Crescent are also located in the map. All dates are in years B.C.

In order to examine the genetic background existing in the first Neolithic communities and its impact over the European genetic pool, we have studied 3 archaeological sites in Syria located in two geographic areas in which agricultural practices were first documented: the middle Euphrates valley and the oasis of Damascus (Figure 1). These sites are dated back to the Pre-pottery Neolithic B period (PPNB). It is during this initial Neolithic phase that animal husbandry first appears, while full-scale agricultural practices are documented in the whole Levant. At the PPNB there is also an increase in the size of the settlements, probably as a response to the population growth derived from the consolidation of the new food-producing economic system [32].

The obtained results allowed us to put into context ancient DNA results from available European Early Neolithic populations, to draft a general model of the Neolithization in Europe and to propose probable routes of expansion of the first Neolithic communities.

## Results

### DNA preservation in ancient Near Eastern Neolithic samples

DNA preservation at the studied samples was assessed at three levels: (1) Estimating the number of copies of the target mtDNA in some of the extracts using a specific Real Time PCR design, (2) estimating the percentage of reproducible Hypervariable Segment I mitochondrial DNA (mtDNA-HVS1) sequences out of all the analyzed samples and (3) computing the miscoding lesions in clone sequences.

The average number of mtDNA HVS1 copies per amplified volume of extract was in all cases higher than 1000, with a mean value of 10.4×10^4^ in Tell Halula and 1.1×10^6^ in Tell Ramad, corresponding respectively to 7.44×10^−5^ and 7.60×10^−4^ ng/µl (Table S1). Reproducible mtDNA sequences could be recovered from 24 out of 112 DNA extracts, corresponding to 15 different skeletons from Tell Halula and Tell Ramad (see Table S2). Differences in sample recovery success ratios could be a result of the strict screening approach used –in which samples displaying more than 2 negative amplification results were discarded (30% of the aDNA extracts)- and of the differences in efficiency between the amplification strategy used in both laboratories. The overall ratio of endogenous DNA recovery for the studied remains was 23.8%.

The average number of miscoding lesions per clone and nucleotide in the studied samples was 0.0078 in Tell Halula and 0.0047 in Tell Ramad. Individual sample variability ranged from 0.0000 (sample H3) to 0.0303 (sample H68) in Tell Halula and from 0.0006 to 0.0101 in Tell Ramad, indicating a differential preservation across the samples (Table S3). Damage values per sample are within the range reported by other authors in samples with similar chronology from temperate environments (La Braña: 0.0116–0.0163; Can Sadurní: 0.0054–0.0632; Chaves: 0.0092–0.0872; Sant Pau del Camp: 0.0000–0.0133).

### Haplotype composition

Reproducible mtDNA HVS1 sequences were obtained from 15 out of 63 skeletons from the archaeological sites of Tell Halula and Tell Ramad (Table 1). The alignments of both the direct sequences and the clones are presented in Table S3. Sequences have been deposited in Genbank (http://www.ncbi.nlm.nih.gov/genbank) with accession numbers KF601411- KF601425.

**Table 1:** Mitochondrial DNA typing of 15 Near Eastern PPNB skeletons.

Site	Skeleton	Haplotype										Haplogroup
		HVS1 (16126–16369)	Coding region									
			7028	12308	14766	10873	10550	12705	10398	10400	4646	
Tell Halula	H3	16293C^b^	**T**	**A**	**T**	**T**	G	**C**				R0
Tell Halula	H4	16311C	**T**	**G**			**G**					K
Tell Halula	H7	16311C	**T**	**G**			**G**					K
Tell Halula	H8	16223T	**T**	**A**	T	**C**	**A**		**A**	**C**		L3
Tell Halula	H70	16356C	**T**	**A**	**T**	**T**	**A**	**T**			**T**	N*
Tell Halula	H68	16294T^b^	**C**									H
Tell Halula	H53	CRS^a^	T	A				C				HV
Tell Halula	H49	16256T	**C**	A			A	C				H
Tell Halula	H25	16224C 16311C	T	**G**			**G**					K
Tell Halula	H28	16311C^b^	T	**G**			**A**					U*
Tell Ramad	R64-4II	16293C^b^	**T**	**A**	**T**		**A**	**C**				R0
Tell Ramad	R65-14	16224C 16311C 16366T	T	**G**	T		**G**					K
Tell Ramad	R69(2)	16293C	**T**	**A**		**T**		**C**				R0
Tell Ramad	R65-C8-SEB	16224C 16311C 16366T	T	**G**								K
Tell Ramad	R65-1S	16224C 16311C 16366T	T	G			**G**					K

Haplotypes represent variable positions relative to rCRS [70].

a Only positions 16,126–16,256.

b Only positions 16,256–16,369.

SNPs validated in more than one extraction are indicated in bold.

In 10 cases it was possible to reconstruct the complete haplotype (nucleotide positions, np 16,126–16,369), while the extent of degradation of the remaining 5 samples only allowed the recovery of partial haplotypes. Nine different haplotypes were identified. Two of them were shared between 2 individuals of Tell Halula (16311C) and among 3 individuals of Tell Ramad (16224C 16311C 16366T). Motif 16293C, though, was present at both sites, pointing at a pre-existing common genetic pool in the region.

### Shared haplotype analysis

The complete haplotypes were compared to a database of 9,821 mtDNA profiles from 59 modern populations from the Near East and South Eastern Europe and 2 Early Neolithic populations from Central Europe (LBK-AVK Neolithic, [24]) and North Eastern Iberia (Cardial/Epicardial Neolithic, [27]) (see Figure S1 and Table S4). Haplogroup affiliation was also considered in the haplotype search.

**Figure 2:**
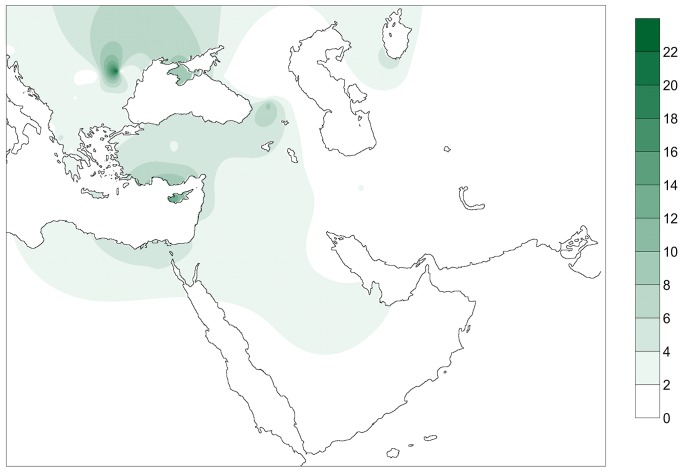
Contour map displaying the percentage of individuals of the database carrying PPNB haplotypes. Only populations with clear geographic distribution were included. Gradients indicate the degree of similarity between PPNB and modern populations (dark: high; clear: small).

The number and percentage of shared haplotypes between the PPNB population and the populations in the database plus the number and percentage of individuals from each population carrying PPNB are presented in Table S5. Figure 2 displays a contour map of the latter built using the same data in a subset of 51 populations.

Two out of the 7 different complete PPNB haplotypes (16356C and 16293C, 28.57% of studied samples) were not represented in any of the modern and ancient populations of the database. From the remaining haplotypes only 16224C 16311C, the basal node of haplogroup K, was shared with the other two ancient populations, displaying a frequency of 9.52% in the Cardial/Epicardial dataset and of 23.08% in the LBK-AVK. This haplotype is distributed nowadays both in South Eastern Europe and the Near East with an average frequency of 4%. However, some populations such as Ashkenazi Jews, Csángó and Cyprus exhibit frequencies of this haplotype higher than 10% (Table S5, Figure 2).

The remaining haplotypes had a very limited geographic distribution, being only documented in 1 individual from Bulgaria (16311C-K); 3 individuals from Turkey, Qatar and Yemen (16223T-L3); 4 Irani, Karakalpak, Turkish and Bedouin individuals (16256T-H) and 3 Druze from Israel (16224C 16311C 16366T-K).

### Haplogroup composition

MtDNA haplogroups could be assigned to 14 out of the 15 skeletons according to the HVS1 sequences obtained and on the diagnostic Single Nucleotide Positions (SNPs) typed following Phylotree rCRS oriented version 15 (Tables 1 and S6).

Haplogroup K was the most prevalent, (*N* = 6, 42.8%) followed by R0 (*N* = 3, 21.42%) and H (*N* = 2, 14.28%). The observed haplogroup frequencies were compared to those of 59 modern populations from the Near East and South Eastern Europe and 2 Early Neolithic populations from Central Europe (LBK-AVK Neolithic, [24]) and North Eastern Iberia (Cardial/Epicardial Neolithic, [27]) (*N* = 11,610) (Table S7).

Haplogroup K was present in almost all populations compared, and its mean frequency in South Eastern Europe and the Near East was around 7%. It reached its highest frequencies in certain populations that have experienced recent population bottlenecks, such as the Askhenazi Jews and the Csángó in Transylvania, Romania [33], [34] and also among Greek Cypriots. Moreover, it was also highly represented in both Cardial/Epicardial (15.56%) and LBK-AVK (23.08%) Early Neolithic datasets. Haplogroup R0 is especially prevalent in the Near East and North Africa with a mean frequency in both regions around 6%. The maximum frequencies of R0 were detected in South Arabian populations such as Bedouin, Oman and Saudi Arabia (Table S7). The rare European haplogroups U* and N* were also detected in 2 individuals in our ancient sample. The mean frequency of haplogroup U* is 2% in the Near East, 0.9% in the Caucasus region and around 1% in Europe, whereas the N* mean frequency is less than 1% in all three datasets. However, both haplogroups reach peaks of frequency in certain populations, such as haplogroup U* in Crete. The case of N* is especially interesting, because apart from Bulgaria, Crete, Romania and Serbia it was only represented in Near Eastern populations (Iran, Jordan, Near Eastern Jews, Oman, Palestine, Saudi Arabia, Syria, Turkmenistan and United Arab Emirates). Moreover, this haplogroup was also detected in 4 Neolithic specimens from Catalonia, in North Eastern Spain, associated to the Cardial/Epicardial culture [27]. Carry- over contamination from these samples processed in the same laboratory can be ruled out, as results were validated in a second independent laboratory.

Finally, the skeleton H8 belonged to the African L3 lineage, this being the most prevalent African haplogroup found in present-day Near Eastern populations.

### Principal Component Analysis and Hierarchical Clustering

Principal Component Analysis with Hierarchical Clustering (PCA-HCA) was used to compare mean haplogroup frequencies of our dataset (Table S7) with the other populations of the database. Details about the method can be found in Table S8.

The first six Principal Components (PCs), accounting for a 90.6% of the variance, were selected for Hierarchical Clustering Analysis. Six clusters (1–6) were defined based on the topology of the hierarchical tree (Figure S2). The decomposition of the inertia computed on 6 axes supported this partition, indicating that with a division in 6 clusters up to an 80% of the data variation could be explained (Table S8). The main haplogroups contributing to the cluster separation were Asian (AS: test value = 12.66; P = 0.000), African (AF: test value = 8.55; P = 0.000), H (test value = 8.96; P = 0.000) and K (test value = 8.01; P = 0.000).

The two biggest groups detected were Clusters 1 and 3, joining 43 of the 60 populations of the database. Cluster 1 mainly included European populations and it was distinguished by high frequencies of haplogroups H, U5, U4 and HV0 and by low frequencies of Asian and African types (Table 2). Near Eastern and some Caucasian datasets were grouped in Cluster 3. They were separated from European populations mainly by high frequencies of haplogroups J and T and low frequencies of H, HV0 and U5. Interestingly, LBK-AVK population was also included in this group. Its similarity with Caucasian populations like Georgia and Chechnya previously suggested by [24] was also evident in our analysis.

**Table 2:** Cluster mean frequencies of each haplogroup.

Haplogroup	Cluster 1	Cluster 2	Cluster 3	Cluster 4	Cluster 5	Cluster 6	Overall
**AS**	1.694−	0.665	3.220−	2.424	41.172+	79.167+	11.055
**H**	39.414+	22.184	16.116−	7.091−	6.312−	0.000−	21.639
**AF**	0.789−	3.209	6.883	52.682+	0.105	0.000	5.629
**K**	5.820	28.887+	6.342	3.524	1.661−	0.000	6.945
**U5**	6.407+	4.253	1.291−	0.000	2.744	0.000	3.243
**HV0**	4.016+	1.083	0.446−	0.000	0.800	1.667	1.732
**HV**	2.552−	1.158	8.041	1.145	17.884+	4.167	6.464
**J**	7.898	5.479	12.619+	6.214	6.384	0.000−	8.795
**T**	9.557	3.506−	11.606+	3.352	5.213−	6.250	8.808
N*	0.169	7.582+	0.737	0.000	0.205	0.000	0.966
**N1**	1.841	2.952	4.086+	6.196+	0.550−	0.000	2.718
**U7**	0.398−	0.575	2.202+	0.202	1.028	0.000	1.134
**U3**	1.968	1.440	4.308+	1.631	0.446−	0.000	2.486
**U1**	1.734	0.635	2.859	0.932	3.785+	0.000	2.205
**U6**	0.169	0.603	0.449	1.190+	0.000	0.000	0.327
**R0**	1.713	5.162	5.382+	5.275	2.470	2.083	3.640
**U4**	2.897+	0.900	1.860	2.365	2.095	0.000	2.082
**U2**	1.232	0.394	1.925	1.661	2.421	3.333	1.697
**R+**	0.678	0.506	1.274+	0.404	0.000	0.000	0.752
**X**	3.428	2.885	2.266	0.651	2.195	0.000	2.494
**U+**	0.333	0.582	0.443	0.325	0.000	0.000	0.334
**W**	2.526	2.620	2.482	0.370	1.487	1.667	2.237
U*	0.962	1.429	1.257	0.202	0.000	0.000	0.902
**I**	1.761	1.314	1.823	2.163	1.044	1.667	1.670
**N2**	0.044	0.000	0.083	0.000	0.000	0.000	0.045

+ Significantly higher haplogroup frequencies (T-test, Sig = 0.05) from the overall haplogroup mean.

− Significantly lower haplogroup frequencies (T-test, Sig = 0.05) from the overall haplogroup mean.

AS: Asian haplogroups, AF: African haplogroups. See Material and Methods for haplogroup grouping.

Cluster 2 included our PPNB sample, grouped together with Ashkenazi Jews, Csángó, Cyprus and Cardial/Epicardial populations. High frequencies of haplogroups K and N* characterized this cluster (Table 2), pinpointing the genetic affinities between the PPNB and the Cardial/Epicardial Neolithic dataset already stressed by the qualitative haplogroup and haplotype analyses.

Cluster 4 included populations from Africa or with a strong African component and it was defined by high frequencies of African haplogroups (L and U6) and low frequencies of haplogroup H. Western Asian populations were clearly separated from Near Eastern datasets in clusters 5 and 6. Both were distinguished by a high frequency of Asian haplogroups and a low frequency of European types. The inclusion of Romani population within cluster 5 is in agreement with its Asian origins [35].

The partition model proposed here supports the existence of geographic barriers for mitochondrial markers. Major geographic zones like Europe, the Near East and Eastern Asia are clearly distinguished. However, populations at boundary zones such as the Caucasus are clustered both with European and Near Eastern pools.

**Figure 3:**
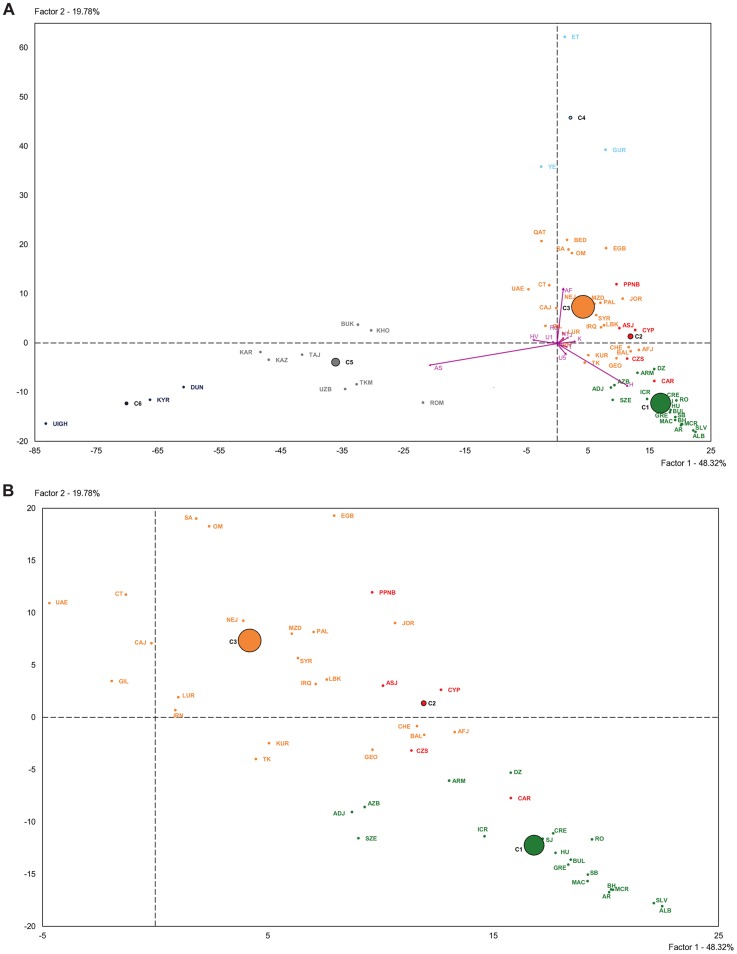
Plot of the two first principal components of the PCA-HCA performed using population haplogroup frequencies. (A) General plot. (B) Zoom plot of Clusters 1, 2 and 3. Population grouping in 6 clusters after HCA is indicated by colors: Cluster 1 (green), Cluster 2 (red), Cluster 3 (orange), Cluster 4 (light blue), Cluster 5 (grey), Cluster 6 (dark blue). Population labels are described in Table S4.

The PCA-HCA for the two first PC factors, accounting respectively for 48.32% and 19.78% total genetic variation, is represented in Figure 3. On one hand, the first PC distinguished populations with and without Asian haplogroups, separating clusters 5 and 6 from 1, 2, 3 and 4. On the other hand, the second PC separated those populations with African (Cluster 4) and non-African (Clusters 1, 2 and 3) haplogroups. Cluster 3, containing Near Eastern and Caucasian populations, occupied an intermediate position in the plot. According to the two first PCs the PPNB population, included in Cluster 2, was equidistant to the centers of this cluster and Cluster 3 and close to modern populations from the Fertile Crescent, such as Jordan and Palestine. Affinities of the PPNB population with populations within Cluster 3 were due to high frequencies of haplogroup R0 in all of them. The Cardial/Epicardial Neolithic population, also member of Cluster 2, was in this case closer to Cluster 1 due to its moderate frequencies of haplogroups H and U5.

Cluster 2 was clearly distinguished from the other 5 clusters by PC4, which summed up a 6.64% of the global genetic variability (Table S8). The graphical plot of PC3 and PC4 separated populations by their frequencies of haplogroups HV, J and T (PC3) and K (PC4) (Figure S3). This graph situated the PPNB sample at the edge of PC4 axis, close to Cardial/Epicardial and Ashkenazi Jew populations.

### Genetic distances

Pairwise F_ST_ genetic distances were computed between the PPNB and the other populations of the database (Table S9). Non-significant pairwise F_ST_ values were obtained between PPNB and Cyprus (F_ST_ = 0.013; P = 0.2734), Ashkenazi Jews (F_ST_ = 0.028; P = 0.1087), Csángó (F_ST_ = 0.022; P = 0.1087) and Khoremian (F_ST_ = 0.0456; P = 0.0805). These populations also exhibited the lowest F_ST_ values. The highest significant distances corresponded to Gilaki, Caucasian Jews and Mazandarian populations (F_ST_>0.2).

When modern populations were grouped in geographic regions, the PPNB population was genetically closer to Near Eastern and Caucasian than to Southern European populations (Table 3). The Cardial/Epicardial and LBK-AVK populations showed low F_ST_ values with the modern Near Eastern pool, as previously stated [24], [27]. It is important to note, however, that the F_ST_ index between LBK-AVK and the pooled Southern European populations was lower than the one reported by [24].

**Table 3:** Pairwise F_ST_ values between PPNB, Cardial/Epicardial, LBK-AVK and pooled modern populations from the Near East, Caucasus, South Asia, North-Eastern Africa and Europe.

	PPNB	LBK-AVK	Cardial	Near East	Caucasus	S Asia	NE Africa	Europe
**PPNB**	*							
**LBK-AVK**	0.08968 (0.01350)	*						
**Cardial**	0.11918 (0.02592)	0.08020 (0.01350)	*					
**Near East**	0.04858 (0.00000)	0.02887 (0.00000)	0.02186 (0.07477)	*				
**Caucasus**	0.05505 (0.01350)	0.05195 (0.00000)	0.02605 (0.08400)	0.02659 (0.0000)	*			
**S Asia**	0.06346 (0.00000)	0.03578 (0.00000)	0.04601 (0.01350)	0.00906 (0.0000)	0.05068 (0.0000)	*		
**NE Africa**	0.08903 (0.00000)	0.05523 (0.00000)	0.00188 (0.38224)	0.02354 (0.0000)	0.03509 (0.0000)	0.03814 (0.0000)	*	
**Europe**	0.07656 (0.00000)	0.02916 (0.00000)	0.05413 (0.00000)	0.00759 (0.0000)	0.04361 (0.0000)	0.00665 (0.0000)	0.03821 (0.0000)	*

Values in brackets indicate P values corrected by the Benjamini-Hochberg method [80].

**Figure 4:**
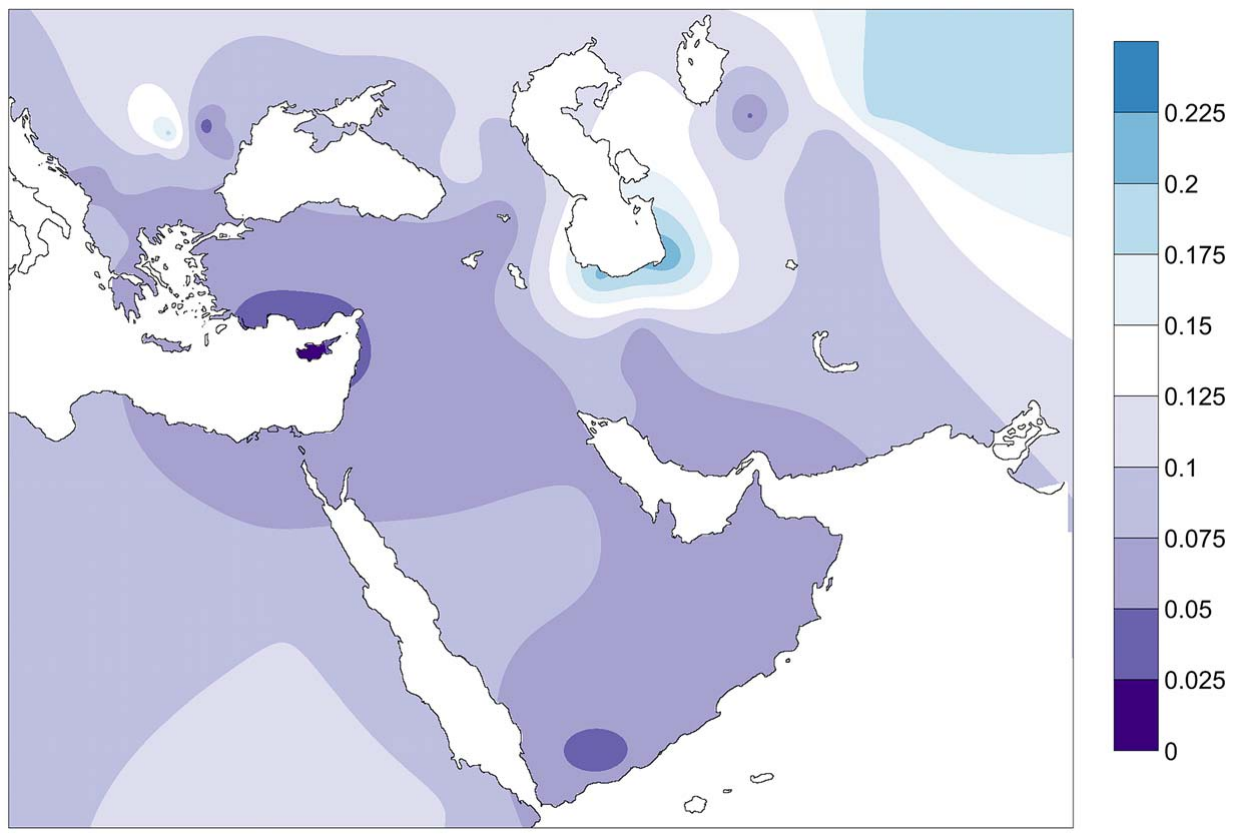
Contour map of Fst distances between the PPNB population and modern populations of the database. Only populations with clear geographic distribution were included. Gradients indicate genetic distance between the PPNB and the modern populations (dark: small; clear: high).

F_ST_ distances between the PPNB and the modern populations were plotted in a contour map (Figure 4). The map showed minimum F_ST_ values in the Fertile Crescent area (Northern Egypt, Palestine, Jordan, Syria and Southern Anatolia) and Cyprus. From this region genetic distances gradually increased westwards across the Balkans, southwards to the Arabic Peninsula and eastwards through the northern Zagros to the Caspian Sea. Peaks of low distance were also detected in the Carpathian basin, Yemen and in North Uzbekistan, South from the Aral Sea.

## Discussion

### Methodology and authenticity of the results

One of the inherent limitations of ancient DNA human studies is the possibility of contamination with exogenous DNA, a risk that is enhanced when human DNA is studied and a PCR approach is used. As a result a series of authentication criteria were proposed early at the beginning of the discipline [36], [37]. However, it has been recognized that on one hand, a complete level of authentication cannot be achieved in most of the cases and on the other, the strict application of all the criteria does not provide a 100% proof of the authenticity of the data [38]. The importance of the retrieved results as a potential comparative framework for other ancient DNA studies requires the reported data to be solid and unambiguous. As such, to guarantee the authenticity of our results we have used a combination of classical criteria of authenticity and a self-interpretative approach as suggested by [38]. These criteria include the replication of the results within the same or in a second laboratory, Real-Time PCR estimation of the number of DNA copies in the extracts, bacterial cloning of amplicons and a self-critical analysis of the obtained results. Trace contaminant DNA was detected through a detailed analysis of clone sequences. Phylogenetic sense observed between HVS1 mtDNA fragments and haplogroup specific SNPs at the mtDNA coding region provided further support to the authenticity of the obtained results. Sequence artifacts like chimerical haplotypes arisen by amplification of fragments of multiple origin (*i.e* contaminant, endogenous and damaged) could be ruled out through replication, as they occur at random and are not reproducible in different amplifications and extractions from the same or different skeletal samples [37]. Moreover, DNA content in the amplified extracts provided in all cases a number of starting copies higher than 1,000, thus making the possibility of displaying hybrid haplotypes highly improbable. The possibility of contamination between samples displaying the same haplotype (*i.e.* H4, H7, H28, H25; H3, R64-4II, R69(2) and R65-14, R65-C8-SEB, R65-1S) could be also discarded as they were processed in different extraction and amplification batches and validated through independent replications, some of them conducted in two different laboratories.

Even though the success recovery ratio is low (23.8%), this study demonstrates that it is feasible to recover ancient DNA genetic information from temperate environments and suggests that other variables rather than the temperature operate in the DNA preservation through several millennia.

Ancient DNA preservation in Near Eastern open-air sites has been previously stated [39]–[42]. The reported success ratios are variable, ranging from 4% [40] to 86% [42]. In the case of Tell Halula, the skeletons were located at opened pits under the main floor of the house. The pits were sealed using a cover made of mud brick of about 20 cm that in some cases was also plastered at the top [43]. This particular burial structure might have protected the human remains from DNA degradation. The absence of sample cleaning with water and the storage in freezers shortly after the excavation, should have also prevented skeletal remains from post-depositional degradation and contamination [39]. The recovery of insoluble collagen fractions (>30,000 Da) in the same remains is also an indicator of their good biomolecular preservation status [44], [45].

### Modern mtDNA Near Eastern variability as a proxy of Near Eastern Neolithic variability

In recent years, the body of ancient DNA data of Neolithic populations has increased dramatically, providing a more accurate picture of local Neolithic dynamics. Some of these studies have also explored the Mesolithic genetic background, interpreting the results in terms of continuity or genetic break with the predecessor hunter-gatherer communities of the area [20], [23], [25], [28]. However, most of the attempts to estimate the Neolithic genetic input in those populations and/or to reconstruct the routes of dispersion of the first farmers into Europe have relied on extant data from modern Near Eastern populations [19], [24], [27], [29]–[31]. In the present research, ancient DNA results from the original human Near Eastern Neolithic communities are presented, to our knowledge, for the first time.

The present study shows that even though the mitochondrial variability of the PPNB population is within the limits of modern Near Eastern, Caucasian and South Eastern European populations (Table 3), both haplotype and haplogroup PPNB frequencies clearly deviate from their modern successors (Figures 2 and 3, Tables S5 and S7). This indicates that the mitochondrial DNA make-up of modern Near Eastern populations may not reflect accurately the genetic picture of the area at the emergence of the Neolithic.

All the detected haplotypes but one -the basal node of haplogroup K- have a null or limited distribution in the modern genetic pool, suggesting that a great bulk of ancient Neolithic lineages were not integrated into their succeeding populations or were erased by subsequent population movements in the region. This is in agreement with previous observations from other Early Neolithic populations [27], [46], and underlines the importance of genetic drift processes at the beginning of the Neolithic [16]. Nevertheless, the multi-population comparative analyses performed here also suggest that certain population isolates of Middle Eastern origin, like the Druze, could have retained an ancient Neolithic genetic legacy through cultural isolation and endogamous practices [47]. Another interesting case are the Ashkenazi Jews, who display a frequency of haplogroup K similar to the PPNB sample together with low non-significant pairwise Fst values, which taken together suggests an ancient Near Eastern origin. This observation clearly contradicts the results of a recent study, where a detailed phylogeographical analysis of mtDNA lineages has suggested a predominantly European origin for the Ashkenazi communities [48]. According to that work the majority of the Ashkenazi mtDNA lineages can be assigned to three major founders within haplogroup K (31% of their total lineages): K1a1b1a, K1a9 and K2a2. The absence of characteristic mutations within the control region in the PPNB K-haplotypes allow discarding them as members of either sub-clades K1a1b1a or K2a2, both representing a 79% of total Ashkenazi K lineages. However, without a high-resolution typing of the mtDNA coding region it cannot be excluded that the PPNB K lineages belong to the third sub-cluster K1a9 (20% of Askhenazi K lineages). Moreover, in the light of the evidence presented here of a loss of lineages in the Near East since Neolithic times, the absence of Ashkenazi mtDNA founder clades in the Near East should not be taken as a definitive argument for its absence in the past. The genotyping of the complete mtDNA in ancient Near Eastern populations would be required to fully answer this question and it will undoubtedly add resolution to the patterns detected in modern populations in this and other studies.

Our PPNB population includes a high percentage (80%) of lineages with a Palaeolithic coalescence age (K, R0 and U*) and differs from the current populations from the same area, which exhibit a high frequency of mitochondrial haplogroups J, T1 and U3 (Table S7). The latter have been traditionally linked with the Neolithic expansion due to their younger coalescence age, diversity and geographic distribution [11], [12], [49]. In addition to the PPNB population, haplogroup T1 is also absent in other Early Neolithic populations analyzed so far [17], [22], [26], [30]. Haplogroup U3 has been found only in one LBK individual and it has been suggested that it could have been already part of the pre-Neolithic Central European mitochondrial background [19].

Haplogroup J is present in moderate frequencies in Central European LBK-AVK populations (11.75%) and it has been proposed as part of the Central European “mitochondrial Neolithic package” [19]. However, it has also been described in one late hunter-gatherer specimen of Germany, raising the possibility of a pre-Neolithic origin [23]. Haplogroup J is present in low frequency (4%) in Cardial/Epicardial Neolithic samples of North Eastern Spain [27], [28], [31]. Absence of Mesolithic samples from the same region prevents making any inference about its emergence during the Mesolithic or the Neolithic. However, its absence in the PPNB genetic background reinforces the first hypothesis.

These findings suggest that (1) late Neolithic or post-Neolithic demographic processes rather than the original Neolithic expansion might have been responsible for the current distribution of mitochondrial haplogroups J, T1 and U3 in Europe and the Near East and (2) lineages with Late Paleolithic coalescent times might have played an important role in the Neolithic expansive process. The first suggestion alerts against the use of modern Near Eastern populations as representative of the genetic stock of the first Neolithic farmers while the second will be explored in depth in the following section.

### Near Eastern Neolithic genetic contribution to the European gene pool

The sharing of mitochondrial haplotypes and haplogroups between pre-pottery farmers from the Fertile Crescent and European Neolithic populations, suggests a genetic contribution of the first Neolithic communities in the European mitochondrial genetic pool.

Haplogroup composition and PCA-HCA of the three ancient datasets compared here allow us to identify K and N*-derived haplogroups as potential Neolithic genetic contributors. Haplogroup K is present in all Early Neolithic datasets published so far with frequencies ranging from 7.7 to 43% (Table S7, [19], [28], [31]). Moreover, it is absent in Central European and Northern Iberian Paleolithic/Mesolithic mitochondrial backgrounds [20], [23], [28]. The presence of “rare” paragroup N* in both Cardial and Epicardial samples from North Eastern Iberia and PPNB populations confirms the connection between both edges of the Neolithic expansion previously suggested in [27].

Haplogroup N1a, representing 12.75% of LBK-AVK samples [19], [24], is not present in our PPNB sample, making it unlikely that this cluster was introduced from the earliest PPNB farmers of this region [23]. A more complex pattern for the LBK-AVK Neolithic expansion route, involving migration and admixture episodes with local hunter-gatherers in frontier zones (for example the predecessor populations of *Starčevo-Criş-Körös* cultures) should be considered in order to explain the available data for Neolithic populations of Central and Northern Europe. To solve this uncertainty, ancient DNA analysis from the Balkans region seems of vital importance.

The signal of both rare N-derived haplogroups over the Neolithic genetic pool must have been erased by subsequent genetic drift events after the consolidation of Neolithic practices, as it has been suggested in other works [15], [27], [50].

### Routes of Neolithic expansion from the Near East into Europe

In the absence of ancient genetic data from populations in the primary and secondary Neolithization areas, a detailed comparison of the genetic composition of the PPNB population with modern adjacent populations can shed light on possible routes of Neolithic expansion.

As for modern Near Eastern populations, the frequency distribution of PPNB mitotypes in modern South Western European populations is limited (see Tables S5 and S7). However, strong genetic affinities at different levels of comparison could be detected with the islands of Cyprus and Crete (Figures 2, 3, 4 and S2, Tables S5, S7 and S9), pointing out at a survival of ancient Neolithic genetic stock in these populations probably through endogamy and geographic isolation.

The absence of an equivalent detectable genetic pattern in modern South-Western Anatolia suggests a primary role of pioneer seafaring colonization through Cyprus and the Aegean islands along the southern coast of Anatolia to the western coast of Greece.

This observation is supported by three facts:

- The archaeological parallels found between the pre-pottery Neolithic of the Levant and those of Cyprus and the Aegean islands in terms of radiocarbon dating, settlement architecture, material culture, cereal and domestic animal species provide evidence for a sea-mediated arrival of Levantine people to Cyprus soon after the development of the agriculture, during the late PPNA or early PPNB, and a further expansion towards the Aegean [51]–[54].
- The finding of PPNB lineages (U*) together with a high frequency of haplogroup K (16%) in a recent survey of Minoan mtDNA indicates a pre-Bronze arrival of these genetic traits of the island. Moreover, this result is in agreement with the archaeological information pointing at a Near Eastern Neolithic origin of the Bronze Age Cretan culture [55].
- Spatial interpolation of radiocarbon dates has identified the Middle Euphrates-South Turkey region as the original centre of Neolithic expansion, and the maritime route through Cyprus, Crete and the Aegean islands as the primary route of Neolithic expansion from the Near East [56].

An alternative scenario of land-mediated expansion through Western Anatolia would assume a survival of the genetic traits observed in the PPNB sample until the end of the period, when Middle-PPNB descendant populations would have expanded to secondary, adjacent areas of Neolithization around 7,500–7,000 years B.C. [57], [58]. This framework is not supported by the obtained data, but cannot be completely discarded as genetic drift or post-Neolithic genetic remodeling of the area might have erased ancient genetic signatures, as already stated from modern Near Eastern populations. Considering that the Neolithic expansion process was not uniform [59], the development of appropriate, spatially-explicit, model-based, statistical inference tools could be of great assistance in fully exploring the probabilities of these and other, competing demographic scenarios.

In conclusion, the study of ancient DNA from the original geographic areas of Neolithic expansion performed here suggests a demic contribution of the first Near Eastern Neolithic in both main European routes of Neolithic expansion. Moreover, the population comparative analysis performed here points out at a leading role of seafaring colonization events in the first Neolithic expansions reaching the European continent. Further ancient DNA data from other primary and secondary areas of Neolithization and new data from frontier zones will be needed to add more resolution over the routes of expansion and the extent and nature of the genetic impact of the Neolithic over the European genetic pool.

## Materials and Methods

### Samples

The studied material consisted of 63 ancient human skeletons from 3 different archaeological sites dating back to the PPNB time period (Table S10 and Figure 1).

Tell Halula is located in the Middle Euphrates basin, 150 Km East of the city of Aleppo in the present territory of Syria. Excavations in the site, 8 hectares in area, have been in progress for the last 18 years by a Spanish Archaeological Mission in Syria. The excavations performed over an area of 2,500 m^2^ documented more than 40 occupation levels with thousands of stratigraphic units. A continuous occupation of the site can be assumed between 7,900–5,700 cal. B.C., spanning from the PPNB to the Neolithic-Chalcolithic transition (Halaf and Obeid periods) [60]–[63]. PPNB occupation phases (I-XX) are located at the southern part of the Tell (sectors 2/4). Each phase is defined by successive human occupations followed by destruction/construction of habitation units. The houses were built one beside the other, oriented southward, and the deceased were buried by digging the graves in the floor of the house and covering them with a slide that allowed a clear association between the graves and the occupation floors. Most of the graves were located at the main entrance of each house, under a porch area. A total of 21 houses from PPNB levels have been unearthed to date, although only 14 of them have documented burial structures [64]. The skeletons analyzed in this paper belonged to occupational phases VIII-XIII on PPNB levels (7,500–7,300 cal. B.C.).

The Tell Ramad archaeological site is located 20 Km south of Damascus on the slopes of Mount Hermon, in a basaltic plateau 830 m in height at the end of the river Wadi Sherkass, which flows in the Damascus basin. Human occupation was documented from early PPNB to ceramic Neolithic [65]. Radiocarbon dating of the site provided dates from 8,300 to 7,750 years B.P. for the PPNB levels (7,300–6,650 cal. B.C.) [66]. The burial model found at Tell Ramad is very similar to that in Tell Halula. The inhumations consisted of narrow tombs in the floor of the house, but evidence of common graves has also been documented.

Dja'de El Mughara is located on the left bank of the Middle Euphrates, also in Syria. The excavations revealed three historical horizons corresponding to Early PPNB (9,400–8,700 B.P., 8,700–8,250 cal. B.C.), Pre-Halaf ceramic Neolithic (7,700–7,400 B.P.) and Early Bronze Age (5,000 B.P.). The burial patterns found at the PPNB levels are very similar to those documented at Tell Halula and Tell Ramad with the graves located under the floor of the houses, but big collective funerary practices were also documented [67], [68]. Most samples from this site were collected by an experienced researcher in ancient DNA analysis (AP-P). The same person selected additional samples during the anthropological analyses.

All the collected samples were neither washed nor treated after excavation. After collection, the samples were sent directly to the laboratory, where they were immediately studied. Contamination prevention measures were taken during all the selection processes, including the use of gloves and face shields. All the researchers involved in the handling of the samples during and after the excavation were typed for mtDNA (Table S11).

### Sample preparation

Whenever possible two samples -preferably teeth- were taken from each individual. Clean and unbroken samples without visible fissures were selected, and then deposited in a sterile container until processed. The surface of all samples was removed with a sandblaster (Base 1 Plus, Dentalfarm) and subsequently UV-irradiated (254 nm) for 30 minutes on all sides. Cleaned samples were finally ground to a fine powder in a cryogenic impact grinder filled with liquid Nitrogen (Spex 6,700).

### Ancient DNA extraction

Approximately 600 mg of the obtained powder was washed a minimum of 3 times with 8 ml 0.5 M EDTA pH 8, and then incubated over-night at 37°C in 10 ml of lysis buffer solution (5 mM EDTA, 10 mM TRIS, 0.5% SDS, 50 µg/ml proteinase K) in a hybridization oven. Tissue remains were removed by centrifugation and DNA was extracted from the supernatant with Phenol/Chloroform. The aqueous phase was concentrated by centrifugation dialysis using Centriplus 30,000 micro-concentrators (Millipore) and desalted with 15 ml sterile water (Braun) to a final volume of 300 µl. Extraction controls without powdered sample were processed in parallel to detect contamination during the extraction process.

### mtDNA amplification and direct sequencing

A region of 305 base pairs (bp) (np 16,095–16,399) of the mtDNA-HVS1 was amplified in the obtained extracts in two overlapping fragments. HVS1 fragment amplification was used as a screening method to detect the presence of amplifiable DNA in the studied samples. Samples were discarded if two consecutive amplifications produced no results.

Two strategies were adopted for the HVS1 *PCR* amplification. In the laboratory at the Universitat de Barcelona, nested-*PCR* reactions using outer and inner primers (Table S12) were performed in a final volume of 25 µl with 5 µl of DNA extract, 1X *Taq* Expand High Fidelity *PCR* buffer (Roche), 2 mM MgCl_2_ (Roche), 0.2 mM dNTP mix (Biotools), 0.4 µM of each primer and 0.06 U of *Taq* Expand High Fidelity (Roche). Nested amplification reactions were subjected to 30 cycles (first reaction) and 40 cycles (second reaction from the first amplified DNA solution) in a Perkin Elmer TC1 Thermocycler (94°C 60 s, 52°C 60 s and 72°C 60 s), with an initial denaturation step at 94 °C for 5 min and a final elongation step at 72 °C for 5 min. In the laboratory at Universidad Complutense de Madrid, one-round *PCR* reactions were set up in a final volume of 25 µl using the Multiplex *PCR* Kit (Qiagen) (5 µl of DNA extract, 1X Multiplex *PCR* Kit (Qiagen) and 0.2 µM of each outer primer). This kit has proven to be extremely efficient for the amplification of ancient DNA [27], [69].

In this case, the cycling conditions using an Eppendorf Mastercycler consisted of 40 cycles of 30 s at 95°C, 90 s at 54°C and 60 s at 72°C, with a previous activation cycle of 15 min at 95°C and a final extension cycle of 10 min at 72°C. Amplicons were visualized in a 2% agarose gel stained with Ethidium Bromide and purification was performed directly from the amplification reaction using the Qiagen *PCR* purification Kit according to the manufacturer's instructions.

Sequencing reactions were performed with the Dye-Terminator Cycle Sequencing Reaction Kit vs 1.2 (Applied Biosystems, Darmstadt, Germany) with one internal primer (L16125, H16259, L16257, H16370). Six microlitres of the *PCR* product were added to a final volume of 10 µl containing 3 µl of the kit and 16 pmol of the selected primer. Cycling sequencing was performed in an Eppendorf Mastercycler according to the supplier's recommendations. Amplification products were analyzed on an automated sequencer ABI PRISM™ 310 (Applied Biosystems, Darmstadt, Germany).

A detailed account of the extractions and amplifications performed can be seen in Table S2.

MtDNA coding regions containing diagnostic SNPs were amplified at the Universidad Complutense de Madrid in monoplex reactions using primers described in Table S12 and the Multiplex *PCR* Kit (Qiagen) (5 µL of DNA, 1X Multiplex *PCR* Kit (Qiagen) and 0.2 µM of each primer). The cycling conditions using an Eppendorf Mastercycler consisted on 40 cycles of 30 s at 94°C, 90 s at 50–54°C (see Table S12) and 60 s at 72°C, with a previous activation cycle of 15 min at 95°C and a final extension cycle of 10 min at 72°C. *PCR* products were purified and sequenced as it has been described above. Haplogroup diagnostic SNPs were typed at least in two separate extracts from the same skeleton in all the cases with the exception of skeleton H53 (Table S6).

### Cloning and sequencing

Only consistent HVS1 amplifications displaying the same mutation pattern between different extractions and PCRs were cloned using the pGEM-T Easy Vector System (Promega). *PCR* products were first incubated for 30 min with 0.2 mM dATP, 1X *PCR* buffer, 2.5 mM MgCl_2_ and 0.1 U/µl *Taq* polymerase at 70°C in order to increase the ligation ratio. Three microlitres of the A-tailed products were ligated into pGEM-T Easy vector at 16°C overnight following manufacturer's recommendations. Five microlitres of the ligation product were transformed into 100 µl of competent cells and the mixture directly plated on IPTG/X-Gal agar plates. Clones carrying *PCR* insert were selected by colony-*PCR* of white colonies (1X *PCR* buffer, 2 mM MgCl_2_, 0.2 mM dNTPs, 0.4 µM each primer and 1.5 U *Taq* polymerase, all from Biotools) using mitochondrial primers (Table S12). The cycling conditions in an Eppendorf Mastercycler were as follows: 94°C 10 min, followed by 30 cycles of 94°C 60 s, 52°C 60 s and 72°C 60 s, linked to a final extension step of 10 min at 72°C. Positive clones were grown in liquid Luria-Bertani broth and plasmidic DNA was purified using the Jetquick Plasmid Miniprep Spin Kit (Genycell, Granada, Spain). Cloned DNA was sequenced with universal primer SP6 or T7 as described above.

### Sequence analysis and consensus haplotype identification

Direct and clone sequences were aligned to the revised Cambridge Reference Sequence (rCRS, [70]) and differences were computed using the Mutation Surveyor software (Demo version 3.24, SoftGenetics, LLC). Carry-over and cross-contamination were examined by comparing cloning results from the same extraction and amplification batch (Table S3). Consensus haplotypes were established from clone and direct sequences considering mutation reproducibility in different extractions/PCRs, concordance with SNP typing and potential contaminations, following the approach of [27].

### Mitochondrial haplogroup inference

Mitochondrial haplogroups were assigned to the amplified samples considering the information of both the HVS1 and the coding region SNPs according to the rCRS oriented version of PhyloTree Build 15 and Haplogrep [71], [72].

### Estimation of miscoding lesions

The number and type of miscoding lesions per sample were computed from the clone alignments manually in the PPNB sample excluding priming sites. The values were normalized by dividing for the number of clones per PCR and the number of sequenced base pairs. Mutations and insertions/deletions within poly-C tracts (positions 16,182–16,193) were not considered.

To provide a frame of comparison for our results, miscoding lesion values were computed in the same way in the clone alignments of two datasets of Mesolithic and Early Neolithic temperate environments [27], [73].

### mtDNA Real Time PCR quantification

A *Taq*-Man Real Time assay was used for the specific quantification of mtDNA HVS1 (np 16,103–16,233) in the obtained extracts using a *Taq*-Man-MGB probe 5′ - AATACTTGACCACCTGTAGTAC (np 16,138-16,159) and primers L16123 (forward) (5′ -ACTGCCAGCCACCATGAATATT, np 16,103–16,123) and H16209 (reverse) (5′ - TGGAGTTGCAGTTGATGTGTGA, np 16,209–16,233). *PCR* reactions were performed using TaqMan Universal *PCR* Master Mix (Applied Biosystems). The samples were loaded onto a standard 96-Well Reaction Plate (Applied Biosystems) and fluorescence detection was performed in a Sequence Detection System ABI Prism 7700 (Applied Biosystems). Four negative controls were included per plate. The DNA concentrations of the extracts were derived from comparison with serial dilutions of a known concentration of human mtDNA standard (10^3^–10^9^ copies equivalent to 3.58×10^−6^ ng/µl and 3.58 ng/µl). All extracts were quantified twice and the average values were considered.

### Precautions and authentication criteria

The following precautions and authenticity standards were observed for validating the obtained results: (1) Samples were collected on the field by staff trained in ancient DNA analysis. (2) Collected samples were unwashed to prevent pre-laboratory contamination. (3) All the analyses were performed in exclusive ancient DNA laboratories in which extraction, preparation of *PCR* reactions and *post-PCR* procedures were physically separated. (4) Access to extraction and *PCR* laboratories was restricted to two researchers (EF and CG), who wore clean-room protective clothes, gloves and facemasks. (5) The laboratories were routinely cleaned with bleach and UV-irradiated. (6) The samples and reagents were manipulated in laminar flow hoods, which were previously cleaned with bleach and irradiated with UV light (254 nm) for 30 minutes. (7) Exclusive material for ancient DNA analysis was employed in every experimental process. (8) Before the analysis, plastic material and pipettes were placed in the cabinet and UV-irradiated for 30 minutes. (9) All individuals were independently extracted at least twice in two independent laboratories except in two cases (see Tables S2, S3). (10) Each studied mtDNA fragment was amplified in separate laboratories at least twice. (11) Only extracts and amplicons from extraction and amplification groups providing negative results at the blanks were considered. (12) Reproducible direct sequences were cloned, and between 10 and 15 clones per amplicon were sequenced (Table S3). (13) The DNA amount in the DNA extracts was estimated by Real Time *PCR* (Table S1), providing in all cases a number of copies higher than 1,000. This result is high enough to guarantee sequence reproducibility [74]. (14) Obtained mtDNA sequences were compared to those from all the archaeologists (MM), anthropologists (AP-P, JA, IO) and researchers (EF, CG, MT, EP) involved in the retrieval or manipulation of the studied samples in order to detect pre-laboratory and laboratory contaminations. For additional security, staff working at the two laboratories involved in the analysis during this period was also typed (DT, JG, EA, AL, CB, JA) (Table S11). (15) Sequences deriving from the same and close extraction and amplification groups were compared to detect carry-over contaminations and non-consistent results were discarded. (16) Phylogenetic sense was observed between retrieved consensus mitochondrial haplotypes and SNP typing of mitochondrial haplogroups.

These criteria not only meet but exceed in different aspects other ancient DNA reports from Neolithic populations [23]–[25], [30], [31].

### Haplotype and haplogroup databases of mtDNA haplotypes of Near Eastern and South Eastern Europe

A database of 9821 mtDNA-HVS1 haplotypes from 59 modern populations from the Near East and South Eastern Europe and 2 Early Neolithic datasets from Central Europe [24] and North Eastern Iberia [27], belonging respectively to LBK-AVK and Cardial/Epicardial Neolithic cultures, was constructed using published data. Sequence alignment tables were transformed into haplotypes using the program “Haplotyper” designed *ad hoc* (Python). Haplotypes were converted into sequences using Haplosearch [75] and used for calculations.

An additional database of haplogroup frequencies was built using published haplogroup data of 11,610 individuals. The same populations used for the haplotype database were included when haplogroups where known. Haplogroup frequencies from other populations not including published haplotypes were also used.

A description of the populations included in both databases is provided in Table S4. Geographic location of the modern populations of the database is shown in Figure S1.

The 95% confident interval was calculated for the frequencies of the mitochondrial haplogroups found in the PPNB sample in the three ancient population datasets (PPNB, Cardial and LBK), in the three modern meta-populations (Near East, SW Europe, Africa and Caucasus) and in the pooled modern population using non-parametric bootstrap with replacement in SPSSvs21.0 [76].

### Shared haplotype analysis

The number and percentage of shared haplotypes between our PPNB population and the other populations in the database, and the number and percentage of individuals in the database carrying PPNB haplotypes, were estimated using the Arlequin software, version 3.5 [77]. Only information contained in the mtDNA fraction analyzed in our ancient population (np 16,126–16,369) was considered.

### Genetic distances

Pairwise F_ST_ genetic distances [78], [79] were computed between our ancient dataset and the populations included in the haplotype database using the Arlequin software version 3.5 [77]. Only the mtDNA fraction analyzed in our ancient population (np 16,126–16,369) was used for comparison. The significance of the genetic distances was tested by permuting the individuals between the populations 10,000 times. P values were adjusted *post-hoc* to correct for multiple comparisons with the Benjamini-Hochberg method [80] as suggested elsewhere [19] using the function *p.adjust* in R [81].

### Contour maps

The percentage of individuals carrying PPNB haplotypes and the percentage of shared haplotypes and pairwise F_ST_ values calculated between the PPNB population and the other populations in the database were graphically plotted in a map using Surfer version 9 (Golden Software). Ethnic groups with disperse geographic location were not considered in the analysis (see Table S4).

### Principal Component Analysis and Hierarchical Clustering

A PCA was performed using basal mtDNA haplogroup frequencies of the populations included in the database (see Table S7). Haplogroups with frequencies >1% in the studied regions were considered: H, HV, I, J, K, T, U*, U1, U2, U3, U4, U5, U6, U7, HV0 (including pre-V, V, HV0b, HV0c), W, X, N*, N1, N2, R0 (former pre-HV). Rare U and R European haplogroups were clustered into two groups: U+: U8, U9 and R+: R1, R2. African and Asian haplogroups were also grouped as follows: African haplogroups (AF): L0-L7, M1; Asian haplogroups (AS): A, B, C, D, E, F, G, M*, M3-48, N9, R5, R9, Q, Y, Z.

HCA was performed over the six first principal components using Euclidean distances and Ward's linkage algorithm [82]. Cluster partitioning was chosen looking at the shape of the obtained Hierarchical tree and examining the *inertia between clusters/total inertia* ratio. In the present study, a partition in 6 clusters was explored. An analysis of the variance was used to evaluate the distances between the clusters.

The statistical program SPAD.N Ver. 5.6 (Système Portable Pour L'Analyse de Donnés), DECISIA, France; [83] was used for both PCA and HCA analyses.

### Accession numbers

The 15 mtDNA sequences reported in this paper have been deposited in Genbank with accession numbers KF601411- KF601425.

## Supporting Information

Figure S1 **Geographic location of modern populations used for phylogenetic and statistical comparisons.** Ethnic groups with unclear or disperse geographic location are not represented. Population labels are described in Table S4.
(TIF)

Figure S2 **Hierarchical tree built using haplogroup frequencies from PPNB, modern and ancient populations from the database.** Cluster partitions are indicated in colors.
(TIF)

Figure S3 **Plot of the third and fourth principal components of the PCA-HCA performed using population haplogroup frequencies.** Population grouping in 6 clusters after HCA is indicated in colors: Cluster 1 (green), Cluster 2 (red), Cluster 3 (orange), Cluster 4 (light blue), Cluster 5 (grey), Cluster 6 (dark blue). Population labels are described in Table S4.
(TIF)

Table S1 **Real time *PCR* quantification results of extracted ancient DNA. Rn: Normalized Reporter; Ct: Threshold cycle. SD: Standard Deviation; CV: Coefficient of Variation.**
(DOCX)

Table S2 **DNA extractions and HVS1 amplifications performed.** The number of amplifications per mtDNA HVS1 is indicated. Fragment 1: HVS1 positions 16,126–16,258. Fragment 2: HVS1 positions 16,258–16,369. Laboratory 1: Universitat de Barcelona, Laboratory 2: Universidad Complutense de Madrid, ^c^ Cloned amplifications. ^*^ Reproducible amplifications not cloned.
(DOCX)

Table S3 **Sequence alignment of direct sequences and clones of the studied samples.** Direct and clone sequences have been aligned to rCRS [70]. Sequences of direct amplifications are presented in bold case and separated from clone sequences by lines. Names for individual sequences are as follows: SKELETON (sample number)-extraction number/mtDNA fragment number/*PCR*number/C followed by the clone number. In direct sequences, the clone number is replaced by “DIR”. Boxes in the reference sequence indicate primer annealing regions. Different types of DNA molecules are highlighted in colors. Pink: Endogenous sequence; Grey: Staff contaminant DNA; Yellow, green and violet: Carry-over contaminant sequences. Miscoding lesions and *Taq* polymerase errors are also highlighted as follows: Blue: Type I miscoding lesions; Green: Type II miscoding lesions; Orange: Other lesions. The last sheet contains the estimated number and percentage of miscoding lesions per position and skeleton.
(XLS)

Table S4 **Description of the 60 modern and 2 ancient Near Eastern and European populations used for comparison.**
(XLSX)

Table S5 **Distribution of the PPNB haplotypes in the populations included in the haplotype database.**
(XLSX)

Table S6 **DNA extractions and coding region SNP amplifications performed.**
(DOCX)

Table S7 **MtDNA haplogroup frequencies of PPNB and populations of the database.** Sheet 1: Absolute frequencies, Sheet 2: Relative frequencies, Sheet 3: 95% confident interval (CI) for haplogroup frequencies found in the PPNB sample, Sheet 4: Plots of haplogroup frequencies in modern and ancient populations from the database. Only haplogroups present in the PPNB, Cardial/Epicardial and LBK populations are displayed. Population labels are described in Table S4.
(XLSX)

Table S8 **Details of the PCA-HCA performed over haplogroup frequencies of the PPNB sample and the other populations of the database. **Population labels are described in Table S4.
(XLSX)

Table S9 **Pairwise F_ST_ values between the PPNB sample and the populations included in the haplotype database.** Sheet 1: F_ST_ values, Sheet 2: P values corrected by the Benjamini-Hochberg method [80].
(XLSX)

Table S10 **Archaeological and anthropological information of the studied samples.** Tooth samples are labeled according to FDI World Dental Federation nomenclature when the type of tooth is known. Other cases are labeled as follows: I: Incisor, C: Canine, P: Premolar, M: Molar. Definitive teeth are labeled in upper-case letters and deciduous teeth in lower-case letters. Dental germs are indicated by “g” before the tooth nomenclature.
(DOCX)

Table S11 **HVS1 mtDNA sequences of the research and laboratory staff involved in sample handling.** Only positions 16,126–16,369 are presented.
(DOCX)

Table S12 **Mitochondrial DNA primers used in this study.**
(DOCX)
