# Supplementary figures and images for: Ancient DNA Analysis of 8000 B.C. Near Eastern Farmers Supports an Early Neolithic Pioneer Maritime Colonization of Mainland Europe through Cyprus and the Aegean Islands
Source: PLoS Genet. 2014 Jun 5;10(6):e1004401. doi: 10.1371/journal.pgen.1004401 (PMC4046922; doi:10.1371/journal.pgen.1004401)

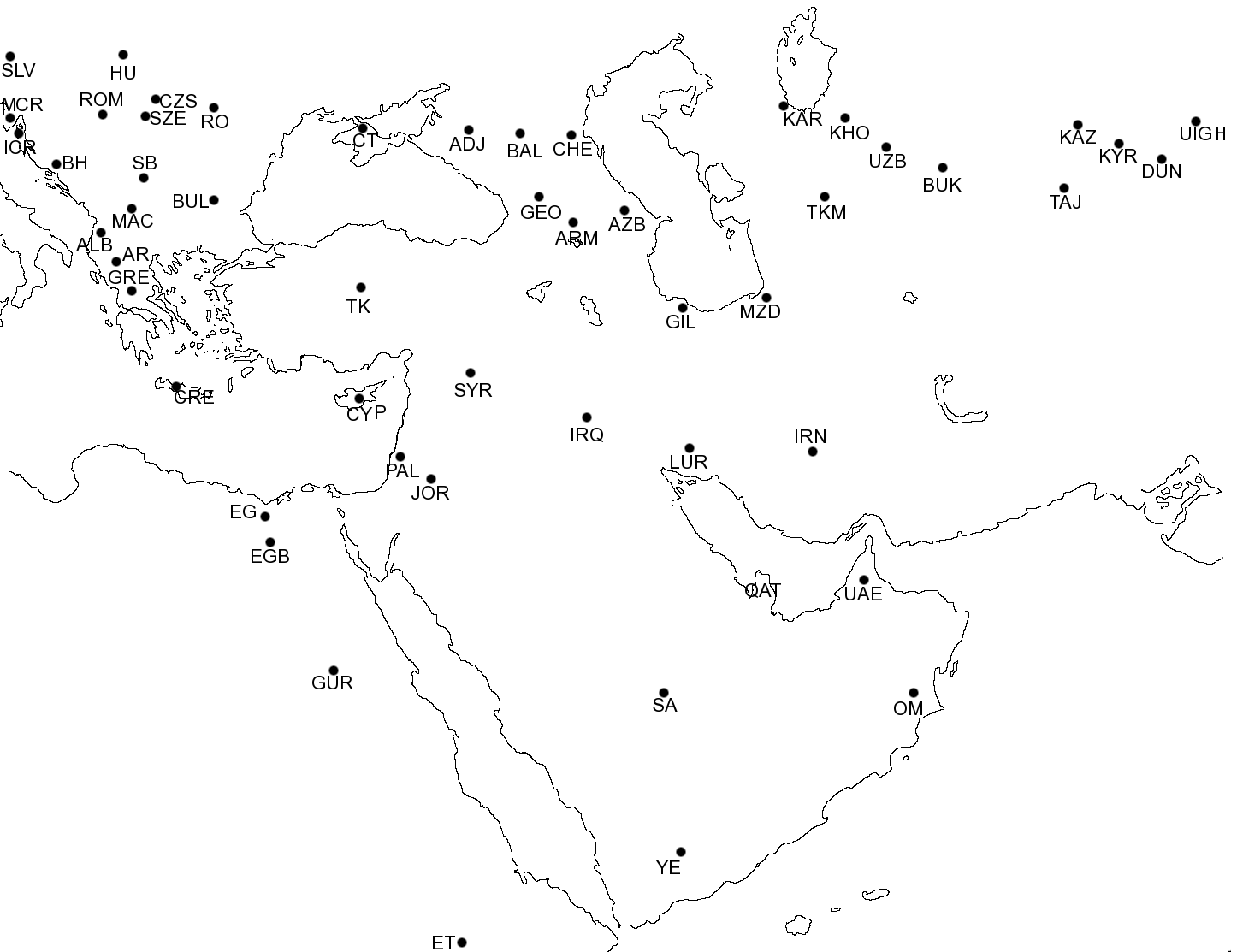

Supplement: Figure S1 — Geographic location of modern populations used for phylogenetic and statistical comparisons. Ethnic groups with unclear or disperse geographic location are not represented. Population labels are described in Table S4. (TIF) [file pgen.1004401.s001.tif]

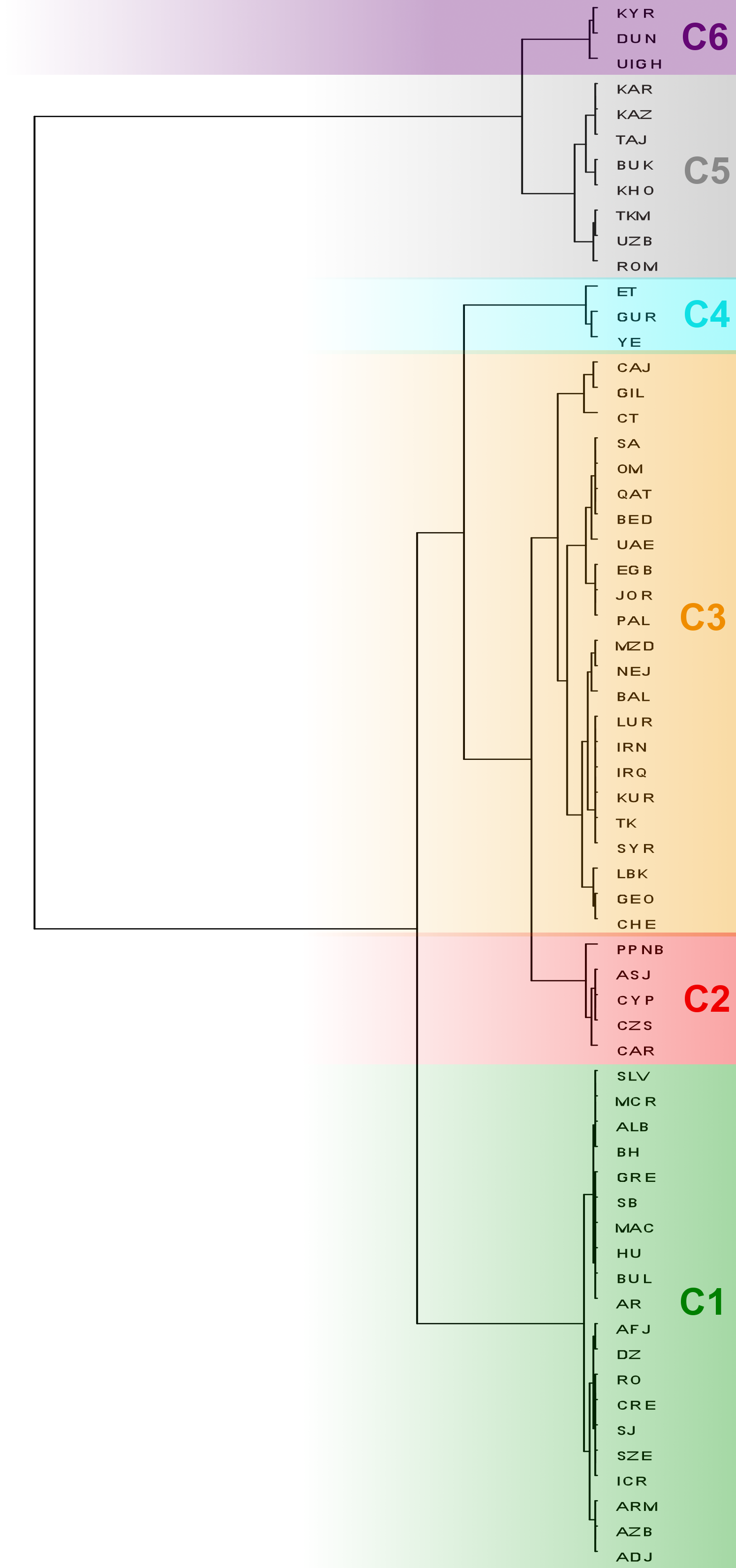

Supplement: Figure S2 — Hierarchical tree built using haplogroup frequencies from PPNB, modern and ancient populations from the database. Cluster partitions are indicated in colors. (TIF) [file pgen.1004401.s002.tif]

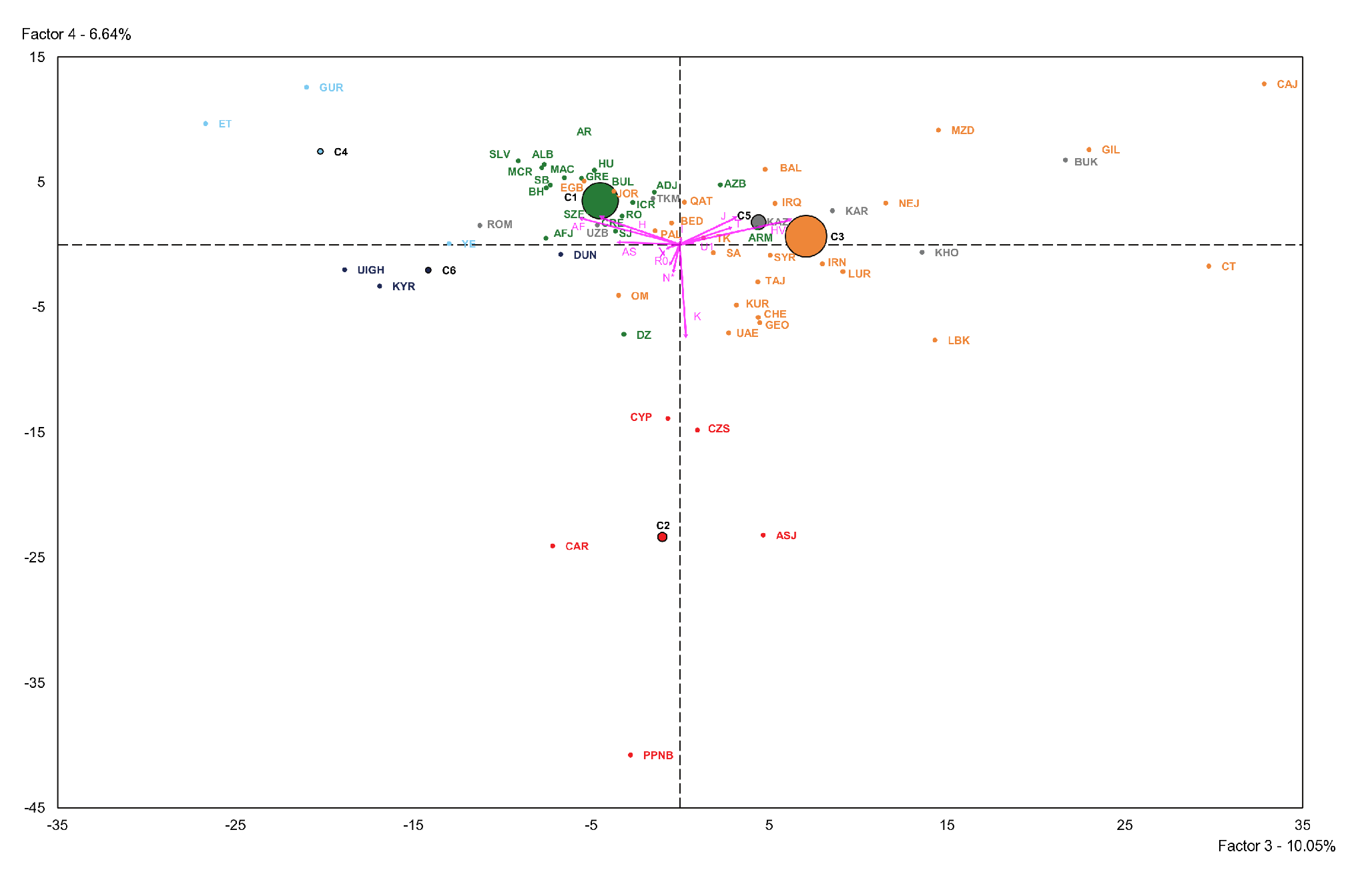

Supplement: Figure S3 — Plot of the third and fourth principal components of the PCA-HCA performed using population haplogroup frequencies. Population grouping in 6 clusters after HCA is indicated in colors: Cluster 1 (green), Cluster 2 (red), Cluster 3 (orange), Cluster 4 (light blue), Cluster 5 (grey), Cluster 6 (dark blue). Population labels are described in Table S4. (TIF) [file pgen.1004401.s003.tif]
