# Supplementary material for: Ancient DNA Analysis of 8000 B.C. Near Eastern Farmers Supports an Early Neolithic Pioneer Maritime Colonization of Mainland Europe through Cyprus and the Aegean Islands
Source: PLoS Genet. 2014 Jun 5;10(6):e1004401. doi: 10.1371/journal.pgen.1004401 (PMC4046922; doi:10.1371/journal.pgen.1004401)
Supplement: Table S1 — Real time PCR quantification results of extracted ancient DNA. Rn: Normalized Reporter; Ct: Threshold cycle. SD: Standard Deviation; CV: Coefficient of Variation. (DOCX) [file pgen.1004401.s004.docx]

| Skeleton/  Sample | Rn | Ct | Estimated copy number | Average copy number | SD | CV (%) | Copies/µl | pg target mtDNA/µl |
| --- | --- | --- | --- | --- | --- | --- | --- | --- |
| H3/1 | 8.15x10^3^ | 36.33 | 2.02x10^5^ | 1.85x10^5^ | 2.46x10^4^ | 13.31 | 3.7x10^4^ | 4.05x10^-5^ |
|  | 7.54x10^3^ | 36.6 | 1.68x10^5^ |  |  |  |  |  |
| H3/2 | 8.54x10^3^ | 36.2 | 7.38x10^4^ | 1.35x10^5^ | 8.68x10^4^ | 64.22 | 2.7x10^4^ | 2.96x10^-5^ |
|  | 1.17x10^4^ | 35.02 | 1.97x10^5^ |  |  |  |  |  |
| H4/1 | 8.59x10^3^ | 36.18 | 2.25x10^5^ | 3.22x10^5^ | 1.37x10^5^ | 42.63 | 6.43x10^4^ | 7.05x10^-5^ |
|  | 1.07x10^4^ | 35.29 | 4.19x10^5^ |  |  |  |  |  |
| H4/2 | 1.04x10^4^ | 35.12 | 1.81x10^5^ | 1.67x10^5^ | 1.95x10^4^ | 11.63 | 3.35x10^4^ | 3.67x10^-5^ |
|  | 9.57x10^3^ | 35.32 | 1.54x10^5^ |  |  |  |  |  |
| H7/1 | 7.9x10^3^ | 36.62 | 5.17x10^4^ | 5.73x10^4^ | 7.88x10^3^ | 13.75 | 1.15x10^4^ | 1.26x10^-5^ |
|  | 8.27x10^3^ | 36.39 | 6.29x10^4^ |  |  |  |  |  |
| H7/2 | 7.21x10^3^ | 36.71 | 4.8x10^4^ | 5.47x10^4^ | 9.44x10^3^ | 17.27 | 1.09x10^4^ | 1.2x10^-5^ |
|  | 7.89x10^3^ | 36.42 | 6.14x10^4^ |  |  |  |  |  |
| H8/1 | 4.2x10^3^ | 38.05 | 6.08x10^4^ | 7.2x10^4^ | 1.59x10^4^ | 22.07 | 1.44x10^4^ | 1.58x10^-5^ |
|  | 4.83x10^3^ | 37.6 | 8.33x10^4^ |  |  |  |  |  |
| H8/2 | 6.43x10^3^ | 36.68 | 4.21x10^4^ | 4.16x10^4^ | 6.24x10^2^ | 1.50 | 8.32x10^3^ | 9.12x10^-5^ |
|  | 6.51x10^3^ | 36.65 | 4.12x10^4^ |  |  |  |  |  |
| H66/1 | 5.8x10^3^ | 37.32 | 1.01x10^5^ | 1.14x10^5^ | 1.73x10^4^ | 15.27 | 2.27x10^4^ | 2.49 x10^-5^ |
|  | 6.74x10^3^ | 37.01 | 1.26x10^5^ |  |  |  |  |  |
| H66/2 | 9.92x10^3^ | 35.45 | 8.53x10^4^ | 9.87x10^4^ | 1.89x10^4^ | 19.17 | 1.97x10^4^ | 2.16 x10^-5^ |
|  | 9.46x10^3^ | 35.79 | 1.12x10^5^ |  |  |  |  |  |
| H65/1 | 9.67x10^3^ | 35.92 | 2.69x10^5^ | 2.39x10^5^ | 4.34x10^4^ | 18.19 | 4.78x10^4^ | 5.23x10^-5^ |
|  | 8.29x10^3^ | 36.29 | 2.08x10^5^ |  |  |  |  |  |
| H70/1 | 1.12x10^4^ | 35.05 | 1.47x10^5^ | 1.51x10^5^ | 6.19x10^3^ | 4.10 | 3.02x10^4^ | 3.31x10^-5^ |
|  | 1.12x10^4^ | 35.12 | 1.55x10^5^ |  |  |  |  |  |
| H70/2 | 8.19x10^3^ | 36.24 | 5.56x10^4^ | 5.72x10^4^ | 2.3x10^3^ | 4.02 | 1.14x10^4^ | 1.25x10^-5^ |
|  | 7.8x10^3^ | 36.31 | 5.88x10^4^ |  |  |  |  |  |
| H68/1 | 6.72x10^3^ | 37.12 | 3.4x10^4^ | 2.61x10^4^ | 1.13x10^4^ | 43.26 | 5.21x10^3^ | 5.71x10^-6^ |
|  | 4.81x10^3^ | 37.88 | 1.81x10^4^ |  |  |  |  |  |
| H64/1 | 8.36x10^3^ | 36.12 | 8.65x10^4^ | 7.57x10^4^ | 1.53x10^4^ | 20.28 | 1.51x10^4^ | 1.66x10^-5^ |
|  | 9.11x10^3^ | 35.77 | 6.48x10^4^ |  |  |  |  |  |
| H54/1 | 8.75x10^3^ | 35.98 | 7.97x10^4^ | 7.63x10^4^ | 4.81x10^3^ | 6.30 | 1.53x10^4^ | 1.67x10^-5^ |
|  | 8.76x10^3^ | 35.87 | 7.29x10^4^ |  |  |  |  |  |
| H49/1 | 6.38x10^3^ | 36.75 | 1.04x10^5^ | 7.14x10^4^ | 4.62x10^4^ | 64.66 | 1.43x10^4^ | 1.57x10^-5^ |
|  | 9.24x10^3^ | 35.54 | 3.88x10^4^ |  |  |  |  |  |
| H56/1 | 5.63x10^3^ | 37.49 | 8.99x10^4^ | 7.71x10^4^ | 1.81x10^4^ | 23.51 | 1.54x10^4^ | 1.69x10^-5^ |
|  | 4.6x10^3^ | 37.97 | 6.43x10^4^ |  |  |  |  |  |
| H45/1 | 8.97x10^3^ | 36.17 | 7.55x10^4^ | 6.71x10^4^ | 1.19x10^4^ | 17.70 | 1.34x10^4^ | 1.47x10^-5^ |
|  | 8.06x10^3^ | 36.47 | 5.87x10^4^ |  |  |  |  |  |
| H47/1 | 1.02x10^4^ | 35.53 | 6.68x10^4^ | 8.61x10^4^ | 2.74x10^4^ | 31.78 | 1.72x10^4^ | 1.89x10^-5^ |
|  | 8.5x10^3^ | 36.09 | 1.05x10^5^ |  |  |  |  |  |
| H37/1 | 8.24x10^3^ | 36.47 | 5.86x10^4^ | 6.8x10^4^ | 1.33x10^4^ | 19.50 | 1.36x10^4^ | 1.49x10^-5^ |
|  | 9.43x10^3^ | 36.14 | 7.73x10^4^ |  |  |  |  |  |
| H57/1 | 8.17x10^3^ | 36.16 | 2.52x10^4^ | 4.39x10^4^ | 2.65x10^4^ | 60.28 | 8.78x10^3^ | 9.63x10^-6^ |
|  | 5.04x10^3^ | 37.28 | 6.26x10^4^ |  |  |  |  |  |
| R65-7I/1 | 7.67x10^3^ | 36.58 | 1.7x10^5^ | 1.4x10^5^ | 4.28x10^4^ | 30.65 | 2.79x10^4^ | 3.06x10^-5^ |
|  | 6.17x10^3^ | 37.21 | 1.09x10^5^ |  |  |  |  |  |
| R63-1/1 | 8.01x10^3^ | 36.51 | 1.78x10^5^ | 2.35x10^5^ | 7.96x10^4^ | 33.93 | 4.69x10^4^ | 5.14x10^-5^ |
|  | 9.17x10^3^ | 35.81 | 2.91x10^5^ |  |  |  |  |  |
| R65(8)/1 | 4.85x10^3^ | 37.64 | 8.1x10^4^ | 8.54x10^4^ | 6.33x10^3^ | 7.41 | 1.71x10^4^ | 1.87x10^-5^ |
|  | 5.26x10^3^ | 37.49 | 8.99x10^4^ |  |  |  |  |  |
| R65(8)/2 | 7.79x10^3^ | 36.5 | 1.8x10^5^ | 1.53x10^5^ | 3.81x10^4^ | 24.95 | 3.05x10^4^ | 3.35x10^-5^ |
|  | 6.65x10^3^ | 37.01 | 1.26x10^5^ |  |  |  |  |  |
| R65-10/1 | 6.03x10^3^ | 37.24 | 1.07x10^5^ | 1.2x10^5^ | 1.77x10^4^ | 14.78 | 2.39x10^4^ | 2.62x10^-5^ |
|  | 6.66x10^3^ | 36.94 | 1.32x10^5^ |  |  |  |  |  |
| R65-10/2 | 8.85x10^3^ | 36.13 | 2.33x10^5^ | 2.27x10^5^ | 7.86x10^3^ | 3.46 | 4.54x10^4^ | 4.98x10^-5^ |
|  | 8.67x10^3^ | 36.2 | 2.22x10^5^ |  |  |  |  |  |
| R65-1/1 | 1.01x10^4^ | 35.5 | 2.02x10^4^ | 6.39x10^4^ | 6.19x10^4^ | 96.78 | 1.28x10^4^ | 1.4x10^-5^ |
|  | 4.25x10^3^ | 37.55 | 1.08x10^5^ |  |  |  |  |  |
| R65-3 I/1 | 9.16x10^3^ | 35.96 | 6.72x10^4^ | 7.06x10^4^ | 4.8x10^3^ | 6.80 | 1.41x10^4^ | 1.55x10^-5^ |
|  | 8.43x10^3^ | 36.08 | 7.4x10^4^ |  |  |  |  |  |
| R65-4II/1 | 7.69x10^3^ | 36.53 | 5.57x10^4^ | 4.42x10^4^ | 1.63x10^4^ | 36.83 | 8.84x10^3^ | 9.69x10^-6^ |
|  | 5.89x10^3^ | 37.17 | 3.27x10^4^ |  |  |  |  |  |
| R65-4II/2 | -3.59x10^2^ | 40 | No results |  |  |  |  |  |
|  | -3.47x10^2^ | 40 | No results |  |  |  |  |  |
| R65-4II/3 | 7.39x10^3^ | 36.95 | 3.95x10^4^ | 5.83x10^4^ | 2.66x10^4^ | 45.67 | 1.17x10^4^ | 1.28x10^-5^ |
|  | 9.2x10^3^ | 36.14 | 7.71x10^4^ |  |  |  |  |  |
| R65-14/1 | 1.24x10^3^ | 39.91 | 3.33x10^3^ | 1.38x10^4^ | 1.49x10^4^ | 107.38 | 2.77x10^3^ | 3.03x10^-6^ |
|  | 4.32x10^3^ | 37.52 | 2.43x10^4^ |  |  |  |  |  |
| R65-C8-SEA/1 | -2.2x10^2^ | 40 | No results |  |  |  |  |  |
|  | -2.04x10^2^ | 40 | No results |  |  |  |  |  |
| R69(2)/1 | 1.12x10^4^ | 35.01 | 6.42x10^4^ | 1.13x10^5^ | 6.89x10^4^ | 61.02 | 2.26x10^4^ | 2.47x10^-5^ |
|  | 7.34x10^3^ | 36.13 | 1.62x10^5^ |  |  |  |  |  |
| R69(2)/2 | 9.78x10^3^ | 35.92 | 2.69x10^5^ | 2.47x10^5^ | 3.17x10^4^ | 12.82 | 4.94x10^4^ | 5.42x10^-5^ |
|  | 8.74x10^3^ | 36.18 | 2.25x10^5^ |  |  |  |  |  |
| R65-C8-SEB/1 | 9.65x10^3^ | 35.67 | 1.14x10^5^ | 1.39x10^5^ | 3.51x10^4^ | 25.27 | 2.78x10^4^ | 3.05x10^-5^ |
|  | 1.08x10^4^ | 35.24 | 1.64x10^5^ |  |  |  |  |  |
| R65-1S/1 | 5.9x10^3^ | 29.68 | 1.04x10^7^ | 2.03x10^7^ | 1.39x10^7^ | 68.72 | 4.05x10^6^ | 4.44x10^-3^ |
|  | 1.81x10^4^ | 28.31 | 3.01x10^7^ |  |  |  |  |  |
| R66-N4-Nº400/1 | 8.36x10^3^ | 36.3 | 6.77x10^4^ | 7.93x10^4^ | 1.64x10^4^ | 20.63 | 1.59x10^4^ | 1.74x10^-5^ |
|  | 9.35x10^3^ | 35.95 | 9.08x10^4^ |  |  |  |  |  |
| SK-A2/1 | 8.45x10^3^ | 36.14 | 6.86x10^4^ | 6.62x10^4^ | 3.41x10^3^ | 5.15 | 1.32x10^4^ | 1.45x10^-5^ |
|  | 8.34x10^3^ | 36.05 | 6.38x10^4^ |  |  |  |  |  |
| SK-D4 16/1 | 7.86x10^3^ | 36.25 | 3.71x10^4^ | 4.77x10^4^ | 1.49x10^4^ | 31.30 | 9.53x10^3^ | 1.04x10^-5^ |
|  | 6.23x10^3^ | 36.8 | 5.82x10^4^ |  |  |  |  |  |
| SK-N4/1 | 4.84x10^3^ | 37.35 | 2.55x10^4^ | 2.46x10^4^ | 1.2x10^3^ | 4.89 | 4.92x10^3^ | 5.39x10^-6^ |
|  | 5.03x10^3^ | 37.26 | 2.38x10^4^ |  |  |  |  |  |
| SK-R3/1 | 6.53x10^3^ | 36.8 | 3.07x10^4^ | 3.4x10^4^ | 4.62x10^3^ | 13.58 | 6.8x10^3^ | 7.45x10^-6^ |
|  | 5.8x10^3^ | 37.03 | 3.73x10^4^ |  |  |  |  |  |
| SK-R3/2 | 4.92x10^3^ | 37.41 | 4.95x10^4^ | 3.6x10^4^ | 1.9x10^4^ | 52.76 | 7.21x10^3^ | 7.9x10^-6^ |
