# Supplementary material for: Ancient DNA Analysis of 8000 B.C. Near Eastern Farmers Supports an Early Neolithic Pioneer Maritime Colonization of Mainland Europe through Cyprus and the Aegean Islands
Source: PLoS Genet. 2014 Jun 5;10(6):e1004401. doi: 10.1371/journal.pgen.1004401 (PMC4046922; doi:10.1371/journal.pgen.1004401)
Supplement: Table S2 — DNA extractions and HVS1 amplifications performed. The number of amplifications per mtDNA HVS1 is indicated. Fragment 1: HVS1 positions 16,126–16,258. Fragment 2: HVS1 positions 16,258–16,369. Laboratory 1: Universitat de Barcelona, Laboratory 2: Universidad Complutense de Madrid, c Cloned amplifications. * Reproducible amplifications not cloned. (DOCX) [file pgen.1004401.s005.docx]

| **Site** | **Skeleton** | **Bone/tooth sample** | **DNA extraction** | **Lab.** | **Fragment/PCR** | **Lab.** |
| --- | --- | --- | --- | --- | --- | --- |
| Dja’de El Mughara | SK-A2 | 1 | 1 | 1 | 1/1, 2/1 | 1 |
|  | SK-D4 16 | 1 | 1 | 1 | 1/1, 1/2, 2/1 | 1 |
|  | SK-N4 | 1 | 1 | 1 | 2/1 | 1 |
|  | SK-R3 | 1 | 1 | 1 | 2/1 | 1 |
|  |  | 2 | 1 | 1 | 2/1 | 1 |
| Tell Halula | H-3 | 1 | 1 | 1 | 1/1 | 2 |
|  |  |  | 2 | 2 | 1/1 | 2 |
|  |  | 2 | 1 | 1 | 1/1, 2/1^c^ | 1 |
|  |  |  | 1 | 1 | 2/2^c^ | 2 |
|  | H-4 | 1 | 1 | 2 | 1/1^c^, 2/1^c^, 2/2^*^ | 2 |
|  |  |  | 2 | 2 | 1/1^c^, 1/2^*^, 2/1, 2/2 | 2 |
|  |  | 2 | 1 | 1 | 1/1, 2/1, 2/2 | 1 |
|  |  |  | 2 | 2 | 1/1^*^, 2/1^*^ | 2 |
|  | H-7 | 1 | 1 | 1 | 1/1^c^, 2/1^*^ | 1 |
|  |  |  | 1 | 1 | 1/2^c^, 2/2^*^ | 2 |
|  |  | 2 | 1 | 1 | 2/1^*^ | 1 |
|  |  |  | 1 | 1 | 1/1^c^ | 2 |
|  |  |  | 2 | 2 | 1/1^c^, 2/1 | 2 |
|  | H-8 | 1 | 1 | 1 | 1/1 | 1 |
|  |  | 2 | 1 | 1 | 2/1^c^ | 1 |
|  |  |  | 1 | 1 | 1/1, 2/2^c^ | 2 |
|  |  |  | 2 | 2 | 1-2/1^c^, 2/1^c^ | 2 |
|  | H-24 | 1 | 1 | 1 | 2/1 | 1 |
|  |  |  | 1 | 1 | 1/2 | 2 |
|  | H-25 | 1 | 1 | 1 | 2/1 | 1 |
|  |  |  | 1 | 1 | 1/1,1/2 | 2 |
|  |  |  | 2 | 2 | 1-2/1^c^, 2/1^c^ | 2 |
|  | H-26 | 1 | 1 | 1 | no results | 2 |
|  | H-28 | 1 | 1 | 1 | 2/1^*^ | 1 |
|  |  |  | 2 | 1 | 2/2^c^ | 2 |
|  | H-30 | 1 | 1 | 1 | no results | 2 |
|  | H-35 | 1 | 1 | 1 | no results | 2 |
|  | H-36 | 1 | 1 | 1 | 2/1 | 1 |
|  |  |  | 1 | 1 | 1/1, 1/2 | 2 |
|  | H-37 | 1 | 1 | 1 | 1/1, 2/1 | 1 |
|  |  |  | 1 | 1 | 1/2 | 2 |
|  | H-43 | 1 | 1 | 1 | 2/1 | 1 |
|  |  |  | 1 | 1 | 1/1, 1/2 | 2 |
|  |  |  | 2 | 2 | 2/1 | 2 |
|  | H-44 | 1 | 1 | 1 | 2/1 | 1 |
|  | H-47 | 1 | 1 | 1 | 1/1, 2/1, 2/2 | 1 |
|  | H-48 | 1 | 1 | 1 | 2/1 | 1 |
|  |  |  | 2 | 2 | 2/1 | 2 |
|  | H-49 | 1 | 1 | 1 | 1/1, 2/1^*^ | 1 |
|  |  |  | 1 | 1 | 1/2^c^ | 2 |
|  |  |  | 2 | 2 | 1/1, 1/2^c^, 2/1^*^ | 2 |
|  | H-53 | 1 | 1 | 1 | 1/1^*^, 2/1 | 1 |
|  |  |  | 1 | 1 | 1/2^c^, 2/2 | 2 |
|  | H-54 | 1 | 1 | 1 | no results | 2 |
|  | H-56 | 1 | 1 | 1 | no results | 1, 2 |
|  | H-57 | 1 | 1 | 1 | 1/1, 2/1, 2/2 | 1 |
|  | H-64 | 1 | 1 | 1 | 1/1 | 1 |
|  |  |  | 2 | 2 | 2/1 | 2 |
|  | H-65 | 1 | 1 | 1 | no results | 1, 2 |
|  | H-66 | 1 | 1 | 1 | no results | 1, 2 |
|  |  | 2 | 1 | 1 | 2/1 | 1 |
|  | H-68 | 1 | 1 | 1 | 1/1, 2/1^c^ | 1 |
|  |  |  | 2 | 2 | 2/1^c^, 2/2^*^, 2/3 | 2 |
|  | H-70 | 1 | 1 | 1 | 1/1^c^,2/1^*^ | 1 |
|  |  |  | 1 | 1 | 2/2^c^ | 2 |
|  |  | 2 | 1 | 1 | 1/1^c^, 2/1 | 1 |
|  | H-71 | 1 | 1 | 1 | no results | 2 |
|  | H-72 | 1 | 1 | 1 | no results | 2 |
|  | H-73 | 1 | 1 | 1 | no results | 2 |
|  | H-74 | 1 | 1 | 1 | no results | 2 |
|  | H-76 | 1 | 1 | 1 | no results | 2 |
|  | H-78 | 1 | 1 | 1 | no results | 2 |
|  |  | 2 | 1 | 1 | no results | 2 |
|  | H-79 | 1 | 1 | 1 | no results | 2 |
|  | H-80 | 1 | 1 | 1 | no results | 2 |
|  | H-82 | 1 | 1 | 1 | no results | 2 |
|  | H-85 | 1 | 1 | 1 | no results | 2 |
|  | H-89 | 1 | 1 | 1 | no results | 2 |
|  | H-90 | 1 | 1 | 1 | no results | 2 |
|  | H-91 | 1 | 1 | 1 | no results | 2 |
|  | H-93 | 1 | 1 | 1 | no results | 2 |
|  | H-98 | 1 | 1 | 1 | no results | 2 |
|  | H-99 | 1 | 1 | 1 | no results | 2 |
|  | H-111 | 1 | 1 | 1 | no results | 2 |
|  | H-121 | 1 | 1 | 1 | no results | 2 |
|  | H-124 | 1 | 1 | 1 | no results | 2 |
|  | H-125 | 1 | 1 | 1 | no results | 2 |
| Tell Ramad | R65-7I | 1 | 1 | 1 | no results | 1, 2 |
|  | R63-1 | 1 | 1 | 1 | no results | 1, 2 |
|  | R65(8) | 1 | 1 | 1 | no results | 1, 2 |
|  |  | 2 | 1 | 1 | no results | 1, 2 |
|  | R65-10 | 1 | 1 | 1 | no results | 1, 2 |
|  |  | 2 | 1 | 1 | 2/1 | 1 |
|  | R65-1 | 1 | 1 | 1 | 1/1, 2/1, 2/2, 2/3 | 1 |
|  | R65-3 I | 1 | 1 | 1 | 1/1, 2/1 | 1 |
|  |  |  | 1 | 1 | 2/2 | 2 |
|  |  |  | 2 | 2 | 1/1, 2/1, 2/2 | 2 |
|  | R65-4II | 1 | 1 | 1 | 1/1, 1/2, 2/1^c^ | 1 |
|  |  | 2 | 1 | 1 | 1/1, 2/1^c^ | 1 |
|  |  |  | 1 | 1 | 1/2 | 2 |
|  |  | 3 | 1 | 1 | no results | 1, 2 |
|  | R65-14 | 1 | 1 | 1 | 1/1^c^, 2/1^c^ |  |
|  |  |  | 1 | 1 | 2/2^c^, 2/3^c^ |  |
|  |  |  | 2 | 2 | 1/1, 1/2, 2/1, 2/2 | 2 |
|  | R65-C8-SEA | 1 | 1 | 1 | no results | 1, 2 |
|  | R69 | 1 | 1 | 1 | 1/1^c^, 2/1^c^ | 1 |
|  |  |  | 1 | 1 | 1/2, 2/2^c^ | 2 |
|  |  |  | 2 | 2 | 1/1^c^, 2/1, 2/2 | 2 |
|  |  | 2 | 1 | 1 | no results | 1, 2 |
|  |  |  | 2 | 1 | no results | 1, 2 |
|  | R65-C8-SEB | 1 | 1 | 1 | 1/1^c^, 2/1^c^ | 1 |
|  |  |  | 2 | 2 | 1/2^c^, 2/2^c^ | 2 |
|  | R65-1S | 1 | 1 | 1 | 1/1^c^, 1/2^c^, 2/1 | 1 |
|  |  |  | 2 | 2 | 1/3^c^, 2/2, 2/3^*^ | 2 |
|  | R66-N4-Nº400 | 1 | 1 | 1 | 1/1, 2/1 | 1 |
|  |  |  | 1 | 1 | 1/2, 2/2 | 2 |
|  |  |  | 2 | 2 | 1/1, 2/1 | 2 |
