# Supplementary material for: Ancient DNA Analysis of 8000 B.C. Near Eastern Farmers Supports an Early Neolithic Pioneer Maritime Colonization of Mainland Europe through Cyprus and the Aegean Islands
Source: PLoS Genet. 2014 Jun 5;10(6):e1004401. doi: 10.1371/journal.pgen.1004401 (PMC4046922; doi:10.1371/journal.pgen.1004401)
Supplement: Table S6 — DNA extractions and coding region SNP amplifications performed. (DOCX) [file pgen.1004401.s009.docx]

| **Skeleton** | **Sample** | **Extraction** | **SNP typing** | | | | | | | | |
| --- | --- | --- | --- | --- | --- | --- | --- | --- | --- | --- | --- |
|  |  |  | **7028** | **12308** | **14766** | **10873** | **10550** | **12705** | **10398** | **10400** | **4646** |
| H3 | 1 | 1 | **T** | **A** | **T** | **T** | G | **C** |  |  |  |
|  |  |  |  |  |  |  |  | **C** |  |  |  |
|  | 2 | 1 | **T** | **A** | **T** | **T** |  | **C** |  |  |  |
| H4 | 1 | 1 | **T** | **G** |  |  | **G** |  |  |  |  |
|  |  | 2 |  |  |  |  | **G** |  |  |  |  |
|  | 2 | 1 | **Y** | **G** |  |  |  |  |  |  |  |
| H7 | 1 | 1 | **T** | **G** |  |  | **G** |  |  |  |  |
|  | 2 | 1 | **T** | **R** |  |  | **G** |  |  |  |  |
|  |  |  | **T** |  |  |  |  |  |  |  |  |
| H8 | 1 | 1 | **T** | **A** | - | **C** | **A** |  | **A** | **C** |  |
|  | 2 | 2 | **T** | **A** | T | **Y** | **A** |  | **A** | **C** |  |
| H70 | 1 | 1 | **T** | **A** | **T** | **T** | **A** | **T** |  |  | **T** |
|  |  |  |  |  |  |  |  | **T** |  |  |  |
|  | 2 | 1 | **T** | **A** | **Y** | **T** | **A** | **T** |  |  | **T** |
| H68 | 1 | 1 | **C** |  |  |  |  |  |  |  |  |
|  |  | 2 | **C** |  |  |  |  |  |  |  |  |
| H53 | 1 | 1 | T | A |  |  |  | C |  |  |  |
| H49 | 1 | 1 | **C** | A |  |  | A | C |  |  |  |
|  |  | 2 | **C** |  |  |  |  |  |  |  |  |
| H25 | 1 | 1 |  | **G** |  |  | **G** |  |  |  |  |
|  |  | 2 | T | **R** |  |  | **G** |  |  |  |  |
| 1H28 | 1 | 1 | T | **R** |  |  | **A** |  |  |  |  |
|  |  | 2 |  | **G** |  |  | **A** |  |  |  |  |
| R65-4II | 1 | 1 | **T** | **A** | **T** |  | **A** | **C** |  |  |  |
| R65-4II | 2 | 1 | **T** | **R** | **T** |  | **A** | **C** |  |  |  |
| R65-14 | 1 | 1 | Y | **G** | Y |  | **R** |  |  |  |  |
|  |  |  |  | **R** |  |  | **G** |  |  |  |  |
| R69(2) | 1 | 1 | **T** | **A** |  | **T** |  | **C** |  |  |  |
|  |  | 2 | **T** | **A** |  | **T** |  | **C** |  |  |  |
| R65-C8-SEB | 1 | 1 | T | **R** |  |  |  |  |  |  |  |
|  |  | 2 |  | **G** |  |  |  |  |  |  |  |
| R65-1S | 1 | 1 | T | G |  |  | **G** |  |  |  |  |
|  |  | 2 |  |  |  |  | **G** |  |  |  |  |
