# Supplementary material for: Ancient DNA Analysis of 8000 B.C. Near Eastern Farmers Supports an Early Neolithic Pioneer Maritime Colonization of Mainland Europe through Cyprus and the Aegean Islands
Source: PLoS Genet. 2014 Jun 5;10(6):e1004401. doi: 10.1371/journal.pgen.1004401 (PMC4046922; doi:10.1371/journal.pgen.1004401)
Supplement: Table S10 — Archaeological and anthropological information of the studied samples. Tooth samples are labeled according to FDI World Dental Federation nomenclature when the type of tooth is known. Other cases are labeled as follows: I: Incisor, C: Canine, P: Premolar, M: Molar. Definitive teeth are labeled in upper-case letters and deciduous teeth in lower-case letters. Dental germs are indicated by “g” before the tooth nomenclature. (DOCX) [file pgen.1004401.s013.docx]

| **Country** | **Site** | **Dating** | **Skeleton** | **Sample** | **Type of sample** | **Archaeological and anthropological information** |
| --- | --- | --- | --- | --- | --- | --- |
| Syria | Dj'ade El Mughara | Early PPNB  (8700-8270 cal. B.C.) | SK-A2 | 1 | 36/37 |  |
|  |  |  | SK-D4 16 | 1 | 13/23 |  |
|  |  |  | SK-N4 | 1 | 61 |  |
|  |  |  | SK-R3 | 1 | 64/65 |  |
|  |  |  |  | 2 | Bone |  |
| Syria | Tell Halula | Middle PPNB  (7500-7300 cal. B.C.) | H3 | 1 | 46 | Excav. 1992. Sector 4. House 4C. Structure E12. Level IX. Collective burial (3 individuals).  Child ~ 6-7 years. Fragmented skeleton. |
|  |  |  |  | 2 | 65 |  |
|  |  |  | H4 | 1 | 11 | Excav. 1992. Sector 4. House 4C. Structure E12. Level IX. Collective burial (3 individuals).  Adult male ~ 20 years. Isolated skull. |
|  |  |  |  | 2 | 48 |  |
|  |  |  | H7 | 1 | 34 | Excav. 1992. Sector 4. House 4C. Structure E14. Level IX. Halulan burial.  Adult female ~ 30 years. Millstone. |
|  |  |  |  | 2 | 48 |  |
|  |  |  | H8 | 1 | 12 | Excav. 1992. Sector 2. House 2A. Structure IIA (A4a?). Secondary burial. Adult unknown sex ~ 20 years |
|  |  |  |  | 2 | 33 |  |
|  |  |  | H24 | 1 | g84 | Excav. 1997. Sector 4. House 4D. Structure E56. Level X. Fetal Halulan burial. Burial goods: sack, shell bracelet. Stone tool.  Child ~ 9-12 months |
|  |  |  | H25 | 1 | g54 | Excav. 1997. Sector 4. House 4D. Structure E59. Level X. Halulan burial. Burial goods: Obsidian blade. Child ~ 2 years. |
|  |  |  | H26 | 1 | Tooth | Excav. 1997. Sector 4. House 4D. Structure E55. Level X. Halulan burial. Burial goods: Shells.  Young adult ~ 14-16 years. |
|  |  |  | H28 | 1 | 64/65 | Excav. 1997. Sector 4. House 4D. Structure E54/E62. Level X. Burial poorly defined. Burial goods: shell belt, obsidian stone, needle.  Child ~ 9 years. |
|  |  |  | H30 | 1 | 13/23 | Excav. 1997. Sector 4. House 4D. Structure E52/E57. Level X. Halulan burial. Burial goods: sack.  Adult male >30 years. |
|  |  |  |  | 2 | 21 |  |
|  |  |  | H35 | 1 | 11 | Excav. 1999. Sector 4. House 4E. Structure E14. Level XI. Burial goods: half shell. Adult female >40 years. Remains without anatomical connection. No burial. |
|  |  |  | H36 | 1 | 26 | Excav. 1999. Sector 4. House 4E. Structure E15. Level XI. Fetal Halulan burial. Burial goods: sack, shroud, half shell. Young adult ~ 13 years. |
|  |  |  | H37 | 1 | 13/23 | Excav. 1999. Sector 4. House 4E. Structure E16. Level XI. Halulan burial. Burial goods: sack and shroud. Young adult ~ 14 years. |
|  |  |  | H43 | 1 | 33/43 | Excav. 1999. Sector 40. Unknown house and level. Burial goods: String of green, read and grey pearls. 6-7 a. Messed remains. |
|  |  |  | H44 | 1 | g74 | Excav. 1999. Sector 40. Unknown house and level. Burial goods: String of green, read and grey pearls.  Child ~ 1 year. Messed remains. |
|  |  |  | H47 | 1 | dI | Excav. 1999. Sector 4. House 4D. Structure E95. Level IX. Fetal burial. Burial goods: 1 shell.  Child ~ 9 months. |
|  |  |  | H48 | 1 | 11 | Excav. 1999. Sector 4. House 4D. Stucture E101. Level IX. Halulan burial. Adult female ~ 20-25 years |
|  |  |  | H49 | 1 | C | Excav. 1999. Sector 4. House 4D. Stucture E93. Level IX. Halulan burial. Burial goods: sack and shroud. Adult female ~ 18 years. |
|  |  |  | H53 | 1 | 32/42 | Excav. 1999. Sector 4. House 4D. Stucture E105. Level IX. Fetal burial. Burial goods: sack and white shroud. Child ~ 10 years. |
|  |  |  | H54 | 1 | 73 | Excav. 1999. Sector 4. House 4D. Structure E104 (connected to E106). Level IX. Halulan burial. Burial goods: 1 flint flake, 1 obsidian flake, 1 butterfly bead.  Child ~ 4 years. |
|  |  |  | H56 | 1 | C | Excav. 1999. Sector 4. House 4D. Stucture E98. Level IX. Halulan burial. Burial goods: Mat, sack and shroud.  Adult male ~ 20 years. |
|  |  |  | H57 | 1 | P | Excav. 1999. Sector 4. House 4D. Stucture E99. Level IX. Halulan burial. Burial goods: Wild boar tusk. Adult male ~ 25 years. |
|  |  |  | H64 | 2 | 18/28 | Excav. 2000. Sector 4. House 4F. Structure E23. Level XII. Halulan burial.  Adult male ~ 18 years |
|  |  |  | H65 | 2 | 16/26 | Excav. 2000. Sector 4. House 4F. Structure E22. Level XII. Halulan burial.  Adult male >40 years. |
|  |  |  | H66 | 1 | dC | Excav. 2000. Sector 4. House 4D. Structure E134. Level VIII. Halulan burial. Flat millstone near the skull.  Child ~ 5 years. |
|  |  |  |  | 2 | dC |  |
|  |  |  | H68 | 1 | M | Excav. 2000. Sector 4. House 4D. Structure E130. Level VIII. Halulan burial. Burial goods: Mat, shroud and possible conical statue.  Adult male ~ 18 years. |
|  |  |  | H70 | 2 | P | Excav. 2000. Sector 4. House 4D. Structure E132. Level VIII. Halulan burial. Burial goods: Mat, mortar pestle, 17 shells, 1 obsidian tool.  Adult female ~ 20 years. |
|  |  |  |  | 3 | 13/23 |  |
|  |  |  | H71 | 1 | 21/22 | Excav. 2002. Sector 4. House 4I. Stucture E22. Level XIII. Halulan burial. Burial goods: 5 pearls, 1 butterfly bead in the right hand.  Adult female >25 years. |
|  |  |  | H72 | 1 | 53 | Excav. 2002. Sector 4. House 4I. Stucture E21. Level XIII. Halulan burial. Burial goods: Mat, shroud. 22 circular pearls, 5 cylindrical pearls and 3 butterfly beads, probably from a necklace. 5 winkles.  Child ~ 3-4 years. |
|  |  |  | H73 | 1 | 21 | Excav. 2002. Sector 4. House 4I. Stucture E23. Level XIII. Halulan burial. Burial goods: mortar pestle made of white limestone.  Adult unknown sex >20 years. |
|  |  |  | H74 | 2 | 17 | Excav. 2002. Sector 4. House 4I. Stucture E24. Level XIII. Halulan burial.  Adult unknown sex ~ 18 years |
|  |  |  | H76 | 1 | g36 | Excav. 2002. Sector 4. House 4H. Structure E53. Level XII. Halulan burial. Burial goods: Shroud.  Child ~ 5-6 years. |
|  |  |  | H78 | 1 | 64 | Excav. 2002. Sector 4. House 4H. Structure E57. Level XII. Double Halulan burial. Burial goods: Sack.  Child ~ 5-6 years. |
|  |  |  |  | 2 | 53 |  |
|  |  |  | H79 | 1 | dC | Excav. 2002. Sector 4. House 4H. Structure E57. Level XII. Double Halulan burial. Burial goods: Sack.  Adult unknown sex ~ 20 years. |
|  |  |  | H80 | 1 | 23 | Excav. 2002. Sector 4. House 4H. Structure E58. Level XII. Double burial.  Young adult male ~ 15 years |
|  |  |  | H82 | 1 | 47/37 | Excav. 2002. Sector 4. House 4H. Structure E61. Level XII.  Adult male >40 years. Only skull. |
|  |  |  | H85 | 1 | C | Excav. 2002. Sector 4. House 4H. Structure E63. Level XII. Halulan burial. Burial goods: Belt made of 26 shells.  Adult unknown sex >40 years. Skull poorly preserved. |
|  |  |  | H89 | 1 | P | Excav. 2002. Sector 4. House 4H. Structure E78. Level XII. Halulan burial.  Child ~ 12 years. Poorly preserved. |
|  |  |  | H90 | 1 | 53 | Excav. 2002. Sector 4. House 4H. Structure E79. Level XII. Halulan burial. Burial goods: Mat.  Child ~ 9 years. |
|  |  |  | H91 | 1 | P | Excav. 2002. Sector 4. House 4H. Structure E81. Level XII. Isolated remains at the bottom of the pit.  Child ~ 12 years. |
|  |  |  | H93 | 1 | C | Excav. 2003. Sector 4. House 4J. Structure E19. Level XIII. Halulan burial. Burial goods: Shroud.  Adult male >25-30 years. |
|  |  |  | H98 | 1 | 42 | Excav. 2003. Sector 2. House 2A. Structure E53. Halulan burial.  Adult female >25 years |
|  |  |  | H99 | 1 | 36 | Excav. 2003. Sector 4. House 4H. Structure E87. Level XI. Destroyed Halulan burial. Burial goods: Sack.  Adult female >25-35 years. |
|  |  |  | H111 | 1 | 18 | Excav. 2003. Sector 4. House 4H. Structure E86. Level XI. Halulan burial.  Adult female >25 years |
|  |  |  | H121 | 1 | P | Excav. 2003. Sector 4. House 4I. Structure E221. Level XII. Halulan burial. Burial goods: Sack, 3 butterfly beds. Adult male ~ 20 years. |
|  |  |  | H124 | 1 | 13 | Excav. 2003. Sector 4. House 4I. Structure E203. Level XII. Halulan burial. Burial goods: Sack, shroud, mat over head. Byblos tip on feet.  Adult male ~ 20 years. |
|  |  |  | H125 | 1 | 37 | Excav. 2003. Sector 4. House 4I. Structure E222. Level XII.  Adult unknown sex >25 years |
| Syria | Tell Ramad | PPNB (7300-6650 cal. B.C.) | R65-7I | 1 | M1 |  |
|  |  |  | R63-1 | 1 | M2 |  |
|  |  |  | R65(8) | 1 | M3 |  |
|  |  |  |  | 2 | P1 |  |
|  |  |  | R65-10 | 1 | dM |  |
|  |  |  |  | 2 | 21 |  |
|  |  |  | R65-1 | 1 | M3 |  |
|  |  |  | R65-3I | 1 | 22 |  |
|  |  |  | R65-4II | 1 | M3 |  |
|  |  |  |  | 2 | 43 |  |
|  |  |  |  | 3 | 33 |  |
|  |  |  | R65-14 | 1 | 42 |  |
|  |  |  | R65-C8SE A | 1 | Tibia |  |
|  |  |  | R69 | 1 | M3 |  |
|  |  |  |  | 2 | 32/42 |  |
|  |  |  | R65-C8SE B | 1 | Long bone |  |
|  |  |  | R65-1S | 1 | Malar bone |  |
|  |  |  | R66 N-4 Nº 400 | 1 | 34/35 |  |
