# Supplementary material for: Ancient DNA Analysis of 8000 B.C. Near Eastern Farmers Supports an Early Neolithic Pioneer Maritime Colonization of Mainland Europe through Cyprus and the Aegean Islands
Source: PLoS Genet. 2014 Jun 5;10(6):e1004401. doi: 10.1371/journal.pgen.1004401 (PMC4046922; doi:10.1371/journal.pgen.1004401)
Supplement: Table S11 — HVS1 mtDNA sequences of the research and laboratory staff involved in sample handling. Only positions 16,126–16,369 are presented. (DOCX) [file pgen.1004401.s014.docx]

|  | **Process** | **Code** | **Haplotype** |
| --- | --- | --- | --- |
| Laboratory stage | Extraction/Amplification | E.F. | CRS |
|  | Extraction/Amplification | C.G. | 16126C, 16294T, 16296T, 16304C |
|  | Post-PCR/Cloning | E.P. | 16294T,16304C,16320C |
|  | Post-PCR/Cloning | M.T. | 16224C, 16311C |
|  | Laboratory staff | D.T. | 16286G, 16362C |
|  | Laboratory staff | J.G. | 16126C, 16294T, 16296T, 16304C |
|  | Laboratory staff | E.A.-P. | 16298C |
|  | Laboratory staff | A.L. | 16218T, 16328A, 16362C |
|  | Laboratory staff | C.B. | 16126C |
|  | Laboratory staff | J.A. | 16193.1C, 16217C, 16274G |
| Pre-laboratory stage | Sample selection | A.P.-P. | 16304C, 16320T |
|  | Anthropologist | P.A. | 16168C |
|  | Anthropologist | I.O. | 16261T |
