# Supplementary material for: Ancient DNA Analysis of 8000 B.C. Near Eastern Farmers Supports an Early Neolithic Pioneer Maritime Colonization of Mainland Europe through Cyprus and the Aegean Islands
Source: PLoS Genet. 2014 Jun 5;10(6):e1004401. doi: 10.1371/journal.pgen.1004401 (PMC4046922; doi:10.1371/journal.pgen.1004401)
Supplement: Table S12 — Mitochondrial DNA primers used in this study. (DOCX) [file pgen.1004401.s015.docx]

| **mtDNA HVRI** | | | | | | | | | |
| --- | --- | --- | --- | --- | --- | --- | --- | --- | --- |
| Primer name | **Sequence 5’-3’** | **Positions** | **Amplified fragment** | | **T annealing** | | **Reference** | | |
| L16112 | CGT ACA TTA CTG CCA GCC | 16095-16112 | 16095-16280  (186bp) | | 53ºC | | Present study | | |
| H16262 | TGG TAT CCT AGT GGG TGA G | 16280-16262 |  |  |  |  | Present study | | |
| L16125 | GCC AGC CAC CAT GAA TAT TG | 16106-16125 | 16106-16276  (171bp) | | 53ºC | | Present study | | |
| H16259 | ATC CTA GTG GGT GAG GGG | 16276-16259 |  |  |  |  | Present study | | |
| L16251 | CAC ACA TCA ACT GCA ACT CC | 16232-16251 | 16232-16399  (168bp) | | 54ºC | | Present study | | |
| H16380 | TCA AGG GAC CCC TAT CTG AG | 16399-16380 |  |  |  |  | Present study | | |
| L16257 | TCA ACT GCA ACT CCA AAG CC | 16238-16257 | 16238-16389  (152bp) | | 54ºC | | Present study | | |
| H16370 | CCT ATC TGA GGG GGG TCA TC | 16389-16370 |  |  |  |  | Present study | | |
| **mtDNA coding region** | | | | | | | | | |
| Primer name | **Sequence 5’-3’** | **Positions** | **Amplified fragment** | **Diagnostic SNP** | | **Haplogroup** | | **T annealing** | **Reference** |
| L6999 | CAA ACT CAT CAC TAG ACA TCG | 6979-6999 | 6979-7086  108bp | 7028C/T | | H/HV | | 55ºC | [24] |
| H7066 | GAA TGA AGC CTC CTAT GAT GG | 7066-7086 |  |  |  |  |  |  | [24] |
| L12227 | GAA AGC TCA CAA GAA CTG C | 12209-12227 | 12209-12357 149bp | 12308A/G | | U/R | | 50ºC | [24] |
| H12341 | GGT TAT AGT AGT GTG CAT GG | 12340-12357 |  |  |  |  |  |  | [24] |
| L14732 | AAA ACC ATC GTT GTA TTT CAA | 14712-14732 | 14712-14811 100bp | 14766C/T | | HV/R | | 55ºC | Present study |
| H14792 | GGA GGT CGA TGA ATG AGT G | 14793-14811 |  |  |  |  |  |  | Present study |
| L10844 | AAT TTG AAT CAA CAC AAC CA | 10825-10844 | 10825-10920 96bp | 10873T/C | | N/L3 | | 55ºC | [63] |
| H14792 | GGG GAA CAG CTA AAT AGG TT | 10901-10920 |  |  |  |  |  |  | [63] |
| 10550F | GCA TTT ACC ATC TCA CTT CTA GG | 10500-10522 | 10500-10628 129bp | 10550A/G | | K/U | | 55ºC | [22] |
| 10550R | GGA GTG GGT GTT GAG GGT TA | 10609-10628 |  |  |  |  |  |  | [22] |
| 12705F | TCA GAC CCA AAC ATT AAT CA | 12664-12783 | 12664-12764  101bp | 12705C/T | | R/N | | 55ºC | Present study |
| 12705R | CGA TGA ACA GTT GGA ATA GG | 12744-12764 |  |  |  |  |  |  | Present study |
| 10398-10400F | AGT CTG GCC TAT GAG TGA CTA C | 10359-10380 | 10359-10444 86bp | 10398A/G-10400C/T | | M/L3 | | 55ºC | Present study |
| 10398-10400R | AAT GAG TCG AAA TCA TTC GTT T | 10423- 10444 |  |  |  |  |  |  | Present study |
| 4646F | AAA TAA ACC CTC GTT CCA CA | 4609-4628 | 4609-4721  113bp | 4646C/T | | U4/U | | 55ºC | Present study |
| 4646R | TTG GTT ATG GTT CAT TGT CC | 4703-4721 |  |  |  |  |  |  | Present study |
